# Systematic review and network meta-analysis of integrated traditional Chinese and conventional medicine for ulcerative colitis

**DOI:** 10.3389/fphar.2026.1785134

**Published:** 2026-06-25

**Authors:** Xue Li, Zhiya Sha

**Affiliations:** Department of Pharmacy, The Affiliated Hospital of Xuzhou Medical University, Xuzhou, Jiangsu, China

**Keywords:** efficacy, integrated traditional Chinese and conventional medicine, network meta-analysis, traditional Chinese medicine decoction, ulcerative colitis

## Abstract

**Objective:**

To optimize clinical management of ulcerative colitis (UC) and evaluate the efficacy of traditional Chinese medicine (TCM), conventional medicine (CM), and integrated TCM- CM therapies.

**Methods:**

Randomized controlled trials (RCTs) investigating integrated TCM–CM treatment for UC were retrieved from PubMed, Embase, Web of Science, CNKI, Wanfang Data, VIP Database, and SinoMed from database inception to June 2025. Study quality was assessed using Review Manager 5.4 and the 7-point Jadad scale. Statistical analyses were performed using Stata MP 15 and R 4.3.1 with Rstudio.

**Results:**

A total of 46 RCTs involving 3,763 patients with UC were included, comprising seven intervention regimens: four commercial Chinese polyherbal preparation (e.g., Yunnan Baiyao) and sixteen classical TCM decoctions (TCMDs; e.g., Baitouweng Decoction). The results showed that conventional medicine combined with TCMD and acupoint catgut embedding achieved the highest total effective rate. CM alone ranked highest in alleviating key clinical symptoms (e.g., diarrhea and abdominal pain) and in reducing inflammatory markers, including IL-8, C-reactive protein (CRP), and erythrocyte sedimentation rate (ESR). For cytokine regulation, comparative advantages varied across interventions: CM + TCMD + acupuncture ranked highest for IL-6 reduction, CM + TCMD ranked highest for IL-10 improvement, whereas CM alone ranked lower for these two outcomes.

**Conclusion:**

Compared with the single use of conventinal medicine, the combined use of traditional Chinese and conventinal medicine in the treatment of ulcerative colitis demonstrates greater overall efficacy. Through network meta-analysis, this study identified optimal intervention regimens for different outcome indicators, including both monotherapy and combination therapy. In clinical practice, the optimal regimens may be selected according to individual patient conditions. Although TCM interventions can improve clinical symptoms and inflammatory biomarkers in patients with ulcerative colitis, relevant clinical score improvement and endoscopic mucosal healing evidence are still insufficient. Further high-quality clinical evidence is still required to confirm the definite clinical efficacy of TCM.

**Systematic Review Registration:**

https://www.crd.york.ac.uk/prospero/display_record.php?RecordID=1109183, identifier CRD420251109183.

## 1 Introduction

Ulcerative colitis (UC) is a chronic inflammatory bowel disease that cannot be cured, as its etiology remains incompletely understood. Clinical manifestations are predominantly abdominal pain and diarrhea accompanied by mucus, pus, and bloody stools, and the disease is characterized by alternating periods of remission and activity. The global prevalence of UC in 2023 was estimated at approximately 5 million cases (Le Berre, Honap and Peyrin-Biroulet, 2023). A cohort study based on the Chinese inflammatory bowel disease database (CHASE-IBD), enrolling a total of 1,081 patients, showed that the cumulative exposure rate among Chinese patients with UC was comparable to that observed in Western countries; however, the surgical rate was lower, and the 10-year cumulative tumor incidence among patients diagnosed with UC was 3.5% (Wan et al., 2024).

The current treatment of UC is primarily aimed at maintaining sustained remission of clinical symptoms and achieving endoscopic healing without the need for corticosteroid therapy (Burri et al., 2020). Therapeutic agents for UC include aminosalicylates, glucocorticosteroids, biologics, and small-molecule targeted agents (Wangchuk, Yeshi and Loukas, 2024). Traditional Chinese medicine (TCM) is characterized by multi-target and multi-pathway therapeutic effects with relatively minimal side effects in the management of UC. In recent years, with the development of TCM, treatment strategies involving TCM alone or integrated traditional Chinese and Western medicine for UC have become increasingly widespread. For example, Gegen Qinlian Decoction, derived from Treatise on Febrile Diseases, can alleviate experimental UC through multiple biological pathways, including regulation of Th2/Th1 and Tregs/Th17 cell balance to correct immune dysregulation, inhibition of NLRP3 inflammasome activation to suppress excessive inflammation, and modulation of the gut microbiota to restore intestinal microecological equilibrium (Hu et al., 2024). Additionally, it can regulate gut microbiota-related tryptophan metabolism and activate the AhR/IL-22 pathway to promote intestinal barrier repair (Wang et al., 2023). Acupuncture and moxibustion promote the recovery of UC by regulating the gut microbiota, mucins (MUC), and immune cells, acting on multiple targets and pathways (Li et al., 2023). As a disease category with recognized advantages for diagnosis and treatment within traditional Chinese medicine, UC requires stronger support from high-quality clinical evidence. Network meta-analysis can quantitatively integrate the results of multiple independent studies, thereby enhancing the strength of the evidence and improving the precision of effect estimates. This approach can also evaluate the effectiveness of different interventions through indirect comparisons, providing a scientific basis for clinical decision-making (Higgins and Welton, 2015). To compare the safety and efficacy of various integrated traditional Chinese and conventional medicine interventions for the treatment of UC, seven intervention strategies were identified: conventional medicine, traditional Chinese medicine decoctions, single Chinese herbs, commercial Chinese polyherbal preparation (CCPP), acupuncture, acupoint catgut embedding, and moxibustion. A network meta-analysis of these interventions was conducted, and relevant randomized controlled trials were systematically reviewed with the aim of optimizing clinical treatment regimens. The categories of intervention methods are detailed in Table 1.

**TABLE 1:** Detailed summary of interventions included in this paper.

Intervention (acronym)	Categories
Conventional medicine	Sulfasalazine
Conventional medicine	Mesalazine
Conventional medicine	Methylprednisolone
Conventional medicine	Metronidazole
Conventional medicine	Olsalazine
Conventional medicine	Bifidobacterium Triple Viable Capsules
Conventional medicine	Balsalazide Sodium
Conventional medicine	Gentamicin
Conventional medicine	Infliximab
Conventional medicine	Dexamethasone
Conventional medicine	Procaine
Traditional Chinese Medicine Decoction (TCMD)	Baitouweng Decoction
Traditional Chinese Medicine Decoction (TCMD)	Gegenqinlian Decoction
Traditional Chinese Medicine Decoction (TCMD)	Clam Shell Powder
Traditional Chinese Medicine Decoction (TCMD)	Gancao Xiexin Decoction
Traditional Chinese Medicine Decoction (TCMD)	Huangqin Decoction
Traditional Chinese Medicine Decoction (TCMD)	Wumei Pill
Traditional Chinese Medicine Decoction (TCMD)	Huanglian Jiedu Decoction
Traditional Chinese Medicine Decoction (TCMD)	Shaoyao Decoction
Traditional Chinese Medicine Decoction (TCMD)	Buzhong Yiqi Decoction
Traditional Chinese Medicine Decoction (TCMD)	Fuzilizhong Decoction
Traditional Chinese Medicine Decoction (TCMD)	Sishen pill
Traditional Chinese Medicine Decoction (TCMD)	Jingjie Lianqiao Decoction
Traditional Chinese Medicine Decoction (TCMD)	Xiaoyao Powder
Traditional Chinese Medicine Decoction (TCMD)	Jianpi Zhixie Decoction
Traditional Chinese Medicine Decoction (TCMD)	Shenling Baizhu Powder
Single Chinese Herb (SCH)	Danshen (*Salvia miltiorrhiza Bge*.Lamiaceae; Dried root and rhizome)
commercial Chinese polyherbal preparation (CCPP)	Bupi Yichang Wan
commercial Chinese polyherbal preparation (CCPP)	Xilei San
commercial Chinese polyherbal preparation (CCPP)	Compound Kushen (Sophora flavescens) Colon-Soluble Capsules
commercial Chinese polyherbal preparation (CCPP)	Yunnan Baiyao Capsules
commercial Chinese polyherbal preparation (CCPP)	Changshu Granules
Acupoint Catgut Embedding (ACE)	​
Acupuncture	​
Moxibustion	​

The detailed ingredients of the herbal decoction are presented in Supplementary Tables S1, S2. Changshu Granules was not prepared by the traditional decoction method of Chinese herbal pieces; therefore, it was classified as a CCPP in this study. The detailed composition is presented in Supplementary Table S1 too.

## 2 Methods

### 2.1 Literature search strategy

This study was registered with the International Prospective Register of Systematic Reviews (PROSPERO) under the registration number CRD420251109183. Studies published from database inception to June 2025 were systematically searched in PubMed, Embase, Web of Science, China National Knowledge Infrastructure (CNKI), Wanfang Data, VIP Database, and SinoMed. The inclusion criteria comprised randomized controlled trials (RCTs) investigating integrated traditional Chinese and conventional medicine (TCM- CM) for the treatment of UC. The categories of interventions covered in this study are detailed in Table 1.

### 2.2 Literature extraction

Two researchers independently extracted raw data and relevant information from the included studies, and any discrepancies were resolved by consensus with a third researcher.

### 2.3 Inclusion and exclusion criteria

Population(P): Patients diagnosed with ulcerative colitis (UC), with no restrictions on region, race, age, or sex.

Intervention(I): Integrated traditional Chinese and conventional medicine (TCM-CM) for UC.

Comparison(C): Conventional biomedicine or other active interventions as used in the included RCTs.

Outcome(O): Total effective rate; Clinical symptoms of UC (abdominal pain, diarrhea, hematochezia, tenesmus); erythrocyte sedimentation rate (ESR), C-reactive protein (CRP); interleukin-6 (IL-6), interleukin-8 (IL-8), interleukin-10 (IL-10), tumor necrosis factor-α (TNF-α).

Study design: randomized controlled trials (RCTs)

Language: Chinese and English.

Exclusion criteria: Animal or *in vitro* studies, duplicate publications, abstracts, meta-analyses, case reports, interventions not meeting inclusion criteria, and studies with unavailable or incomplete data.

### 2.4 Study extraction and quality assessment

Two researchers independently extracted data using Microsoft Excel spreadsheets in accordance with the predefined inclusion and exclusion criteria. The Cochrane Risk of Bias tool version 1 (RoB 1) was applied in accordance with the standards of the Cochrane Handbook for Systematic Reviews of Interventions (version 5.1.0), and Review Manager software (RevMan version 5.4) was used to generate the risk of bias plots. The quality assessment criteria included the following domains: random sequence generation, allocation concealment, blinding of participants and personnel, blinding of outcome assessment, completeness of outcome data, selective reporting, and other potential sources of bias. Each domain was judged as having a low risk, high risk, or unclear risk of bias.

### 2.5 Statistical analysis

Statistical analyses in this study were performed using Stata software. First, a network plot was constructed to visually display the direct and indirect comparison relationships among different intervention measures in the included studies, and the connectivity of the network was evaluated. Subsequently, under the frequentist framework, a random-effects model was adopted to conduct a network meta-analysis (NMA). For binary outcomes, relative risk (RR) with its 95% confidence interval (95% CI) was used as the effect size to estimate the relative efficacy among different intervention measures; for continuous outcomes, the standardized mean difference (SMD) was applied. To evaluate the relative superiority of different interventions, the surface under the cumulative ranking curve (SUCRA) values were calculated for each intervention, and treatment ranking plots were generated. Higher SUCRA values indicate better performance of an intervention across comparisons. Tests of the network consistency assumption included: (1) a loop-specific local inconsistency test; and (2) a global inconsistency test based on the design-by-treatment interaction model. A P value <0.05 was considered to indicate significant inconsistency between direct and indirect evidence in the network, and the results of the corresponding comparisons were interpreted with caution. A league table was generated to present the effect estimates and their confidence intervals for each intervention relative to the reference control. To assess potential publication bias, comparison-adjusted funnel plots were constructed and symmetry was evaluated. Statistical significance was defined as a 95% CI of the RR not including 1, or a 95% CI of the SMD not including 0. In this study, we used the netmeta package in R 4.3.1 to analyze heterogeneity, and the I^2^ statistic was calculated. I^2^ > 50% was considered significant heterogeneity.

## 3 Results

### 3.1 Literature selection and basic characteristics

**FIGURE 1:**
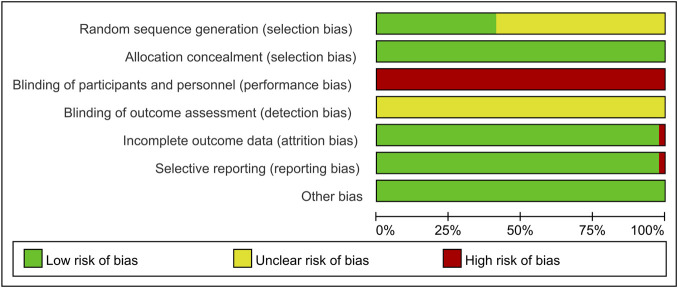
PRISMA flow diagram.

A total of 4,425 records were retrieved through the database searches. After removing duplicate records using the reference management software EndNote, 2,937 records remained. Following title and abstract screening in strict accordance with the inclusion and exclusion criteria, 2,082 records were excluded. After full-text review, a total of 46 studies were finally included in the analysis (Li et al., 2021; Wang, Li, Zhang, Xu and Li, 2006; Bi, 2020; Chang, 2018; Chen et al., 2019; Chen and Zhang, 2017; Deng et al., 2011; Ding et al., 2018; Dong et al., 2023; Du et al., 2022; Fu, 2019; Gu and Luo, 2021; Gu, 2020; Hong et al., 2022; Hu, et al., 2020; Huang, 2019; Huang et al., 2024; Kang et al., 2017; Li et al., 2012; Li et al., 2016; Liu and Zhang, 2024; Liu et al., 2023; Long et al., 2017; Lu et al., 2010; Lu and Gao, 2023; Ma et al., 2021; Qi et al., 2016; Song and Linghu, 2023; Tan et al., 2020; Wang and Li, 2021; Wang, 2014; Wang, 2019; Zhenxing et al., 2022; Wu and Fenglin, 2017; Xu, 2021; Qin et al., 2023; Yang, 2017; Yin, 2013; Lihua et al., 2022; Zhang, 2020; Zhang et al., 2022; Zhang, 2017; Zhang, 2018; Jia et al., 2009; Zhong and Yu, 2023; Zhou et al., 2004). The literature screening process is shown in Figure 1.

The basic characteristics of the included studies are shown in Table 2. A total of 3,763 participants were enrolled, including 1,776 patients in the conventional medicine group, 153 patients in the TCM group, and 1,835 patients in the TCM-CM group. In the CM group, the drugs used in the control group and the corresponding number of studies are as follows: 15 studies used sulfasalazine as the control group, 26 studies involved mesalazine, 1 study used olsalazine as the control group (Li et al., 2012), 1 study adopted balsalazide sodium (Wang, 2014), and 1 study used sulfasalazine combined with methylprednisolone as the control group (Chen et al., 2019); in addition, 2 studies involved metronidazole in the control group (S. Wang, Li, Zhang, Xu and Li, 2006; Kang et al., 2017), 2 studies involved Bifidobacterium Triple Viable Capsules (Liu et al., 2023; Jun Ma, 2021), and 1 study used infliximab as the control group (Tan et al., 2020). The duration of the studies ranged from 2 to 12 weeks. With respect to study populations, all studies primarily included patients diagnosed with UC. The mean age of participants ranged from 31.52 to 50.06 years, and female patients accounted for 44.9% of the total sample. The interventions covered commonly used aminosalicylic acid (5-aminosalicylic acid, 5-ASA) anti-inflammatory agents for the treatment of UC, as well as widely applied TCM formulas based on TCM theory, including Gegen Qinlian Decoction, Shaoyao Decoction, Baitouweng Decoction, and Wumei Pill. In addition, fopur types of CCPP were included: Yunnan Baiyao Capsules, Bupi Yiqi Pills, Xilei Powder, Compound Kushen Colon-Soluble Capsules.

**TABLE 2:** Basic clinical characteristics of all included studies.

First author/Year	Sample size		Average age (Years)		Intervention (T/C)	Course of treatment (weeks)	Outcome indicator	Jadad scale score
	C	T	C	T	​	​	​	​
Bi (2020)	35	35	35.28 ± 2.15	35.24 ± 2.06	CM/CM + TCMD + acupuncture	4	①	4
Chang (2018)	38	40	41.20 ± 3.00	41.20 ± 3.00	CM/CM + TCMD	4	①⑤	3
Chen et al. (2019)	30	30	44.01 ± 6.21	43.88 ± 5.93	CM/CM + CCPP	8	①	4
Chen and Zhang (2017)	50	50	38.65 ± 5.28	38.72 ± 5.21	CM/CM + TCMD	4	②⑤⑥⑧	4
Deng et al. (2011)	30	30	39.58 ± 11.75	42.58 ± 122.25	CM/CM + SCH	8	①⑦	3
Ding et al. (2018)	45	45	26.50 ± 11.00	36.34 ± 3.28	CM/CM + TCMD	4	①⑤⑧	5
Dong et al. (2023)	30	30	44.80 ± 9.80	45.50 ± 10.20	CM/CM + TCMD	12	①②③④	3
Du et al. (2022)	40	40	44.98 ± 2.41	45.12 ± 2.42	CM/CM + TCMD	8	①	4
Fu (2019)	50	50	46.20 ± 2.40	46.90 ± 2.40	CM/CM + TCMD	4	①⑤⑥⑦	3
Lu and Gao (2023)	31/33	34	37.90 ± 7.60 38.20 ± 7.90	37.5 ± 8.20	CM/TCMD + ACE/CM + TCMD + ACE	12	①③⑤⑦	4
Gu and Luo (2021)	30	30	50.29 ± 7.09	49.17 ± 6.52	CM/CM + TCMD	4	①②③④⑥	4
Gu (2020)	30/30	30	45.10 ± 10.52 44.10 ± 12.52	37.44 ± 10.21	CM/TCMD/CM + TCMD + acupuncture	8	①②③④⑦⑧	4
Hong et al. (2022)	34	34	38.16 ± 7.31	38.61 ± 7.15	CM/CM + TCMD	12	②③④	4
Hu, et al. (2020)	40	40	44.69 ± 12.82	44.32 ± 12.56	CM/CM + TCMD	4	②	4
Huang (2019)	50	50	41.84 ± 9.45	42.05 ± 9.23	CM/CM + TCMD	8	①③⑦	4
Huang et al. (2024)	30	30	48.23 ± 5.27	47.18 ± 4.69	CM/CM + TCMD	4	①②⑧	3
Kang et al. (2017)	40	40	39.60 ± 9.40	41.8 ± 8.60	CCPP/CM + CCPP	4	①	3
Wang and Li (2021)	53	54	36.25 ± 4.12	35.78 ± 3.99	CM/CM + TCMD	4	①⑤⑥⑧	4
Li et al. (2012)	35	35	47.66 ± 12.21	45.47 ± 11.85	CM/CM + TCMD	8	①③④	3
Li et al. (2016)	20/20	20	36.95 ± 9.75 36.95 ± 9.35	37.25 ± 10.02	CM/CCPP/CM + CCPP	4	①⑤⑥⑦	3
Liu and Zhang (2024)	51	51	39.49 ± 6.21	39.96 ± 5.83	CM/CM + TCMD	4	①②⑤⑥⑦	4
Liu et al. (2023)	48	50	41.98 ± 7.30	42.31 ± 7.34	CM/CM + TCMD	4	①②⑤⑦	4
Long et al. (2017)	47	47	37.90 ± 4.10	38.20 ± 4.70	CM/CM + ACE	4	①③④	3
Lu et al. (2010)	30/30	30	37.37 ± 10.39 37.13 ± 9.720	44.03 ± 11.70	CM/TCMD/CM + TCMD	8	①⑤⑥⑦	3
Ma et al., 2021	39	39	38.12 ± 1.43	37.92 ± 1.56	CM/CM + TCMD	2	①⑤⑦⑧	4
Qi et al. (2016)	83	82	38.80 ± 13.40	37.90 ± 12.80	CM/CM + TCMD	8	①⑤⑧	4
Song and Linghu (2023)	18	18	39.97 ± 8.70	40.04 ± 7.80	CM/CM + TCMD	4	①②⑤	3
Tan et al. (2020)	34	34	40.67 ± 10.75	41.55 ± 9.08	CM/CM + TCMD	12	①②⑤⑦⑧	4
Wang (2014)	60	62	47.15 ± 6.23	45.26 ± 5.08	CM/CM + TCMD	4	①⑤⑥	4
Wang (2019)	42	42	36.83 ± 2.31	36.17 ± 2.48	CM/CM + TCMD	4	⑧	5
Zhenxing (2022)	59	59	48.63 ± 5.21	48.81 ± 5.03	CM/CM + TCMD	4	⑤⑧	4
Wu and Fenglin (2017)	30	30	45.50 ± 14.50	45.60 ± 15.70	CM/CM + TCMD	8	①②③④	3
Xu (2021)	44	44	40.50 ± 6.84	41.30 ± 6.91	CM/CM + TCMD	12	①⑤⑥	3
Qin et al. (2023)	58	58	46.09 ± 5.01	46.31 ± 5.03	CM/CM + TCMD	8	①②	4
Yang (2017)	37	37	38.14 ± 7.22	38.56 ± 7.48	CM/CM + TCMD	8	①⑤⑦⑧	4
Yin (2013)	30	30	33.10 ± 7.75	33.50 ± 7.50	CM/CM + TCMD	2	①③	3
Lihua (2022)	30	30	44.20 ± 12.20	43.60 ± 11.80	CM/CM + TCMD	8	①③④	4
Zhang (2020)	54	54	44.37 ± 4.06	44.32 ± 4.02	CM/CM + TCMD	4	①③⑤	4
Zhang et al. (2022)	30	30	42.17 ± 10.09	43.60 ± 12.22	CM/CM + TCMD	4	①②③④⑤⑦⑧	3
Zhang (2017)	34	34	27.40 ± 4.90	28.70 ± 5.40	CM/CM + CCPP	4	①④	4
Zhang (2018)	49	49	42.54 ± 6.42	41.86 ± 6.37	CM/CM + TCMD	4	①②⑤	4
Jia et al. (2009)	12	22	31.73 ± 11.58	32.51 ± 12.46	CM/CM + CCPP	8	①	3
Zhong and Yu (2023)	31	31	38.19 ± 3.27	39.01 ± 3.40	CM/CM + TCMD	4	①②③	4
Zhou et al. (2004)	60	60	39.60	40.3	CM/CM + CCPP	2	①	3
Li et al. (2021)	35	35	67.23 ± 5.48	66.94 ± 6.15	CM/CM + TCMD	12	①	4
Wang et al. (2006)	30	30	28–54	27–53	CM/CM + moxibustion	4	①	3

C: control group; T: Experimental group; ① effective rate; ② core clinical manifestations; ③ CPR; ④ ESR; ⑤ TNF-α; ⑥ IL-8; ⑦ IL-6; ⑧ IL-10.

### 3.2 Assessment results of quality

**FIGURE 2:**
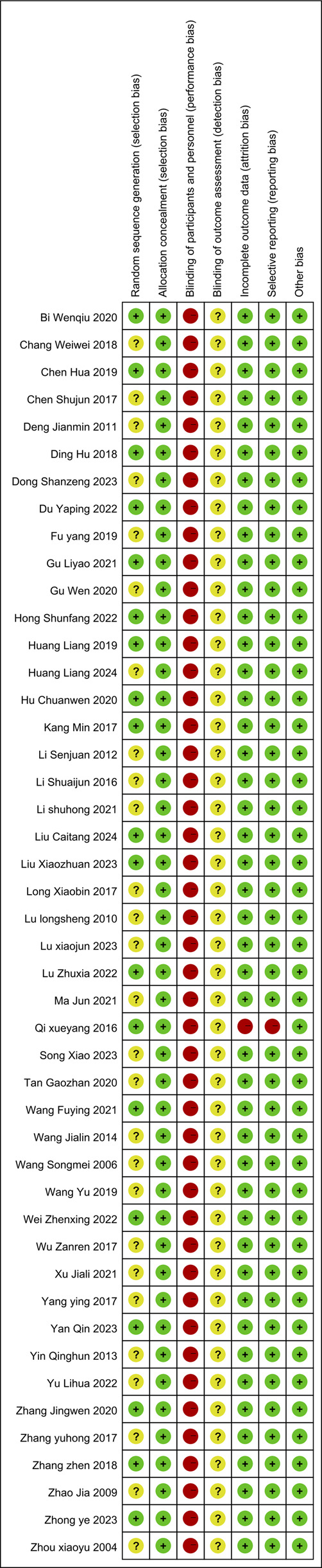
**(a)** Risk of bias summary **(b)** Risk of bias graph.

Of the 46 included RCTs, 18 studies (Bi, 2020; Chen et al., 2019; Yaping Du, 2022; Gu and Luo, 2021; Shunfang Hong, 2022; Hu et al., 2020; Huang, 2019; Kang et al., 2017; Fuying Wang, 2021; Liu and Zhang, 2024; Liu et al., 2023; Xueyang Qi et al., 2016; Zhenxing, 2022; Qin et al., 2023; Jingwen Zhang, 2020; Zhang et al., 2022; Zhang, 2018; Zhong and Yu, 2023) used a random number table method, and one study (Ding et al., 2018) used a random envelope method; these studies were assessed as having a low risk of bias. The remaining studies reported the use of “randomization” but did not provide detailed descriptions of the randomization procedures; therefore, their risk of bias was rated as unclear. In one study (Qi et al., 2016), the number of participants initially enrolled did not correspond to the number of participants included in the outcome analysis, and no explanation was provided for this discrepancy; consequently, the risk of bias was assessed as high. No other studies reported loss to follow-up or participant dropout, and no attrition bias was identified. The individual and overall risk of bias assessments of the included studies are presented in Figure 2.

### 3.3 Effective rate: evidence network and network meta-analysis

**FIGURE 3:**
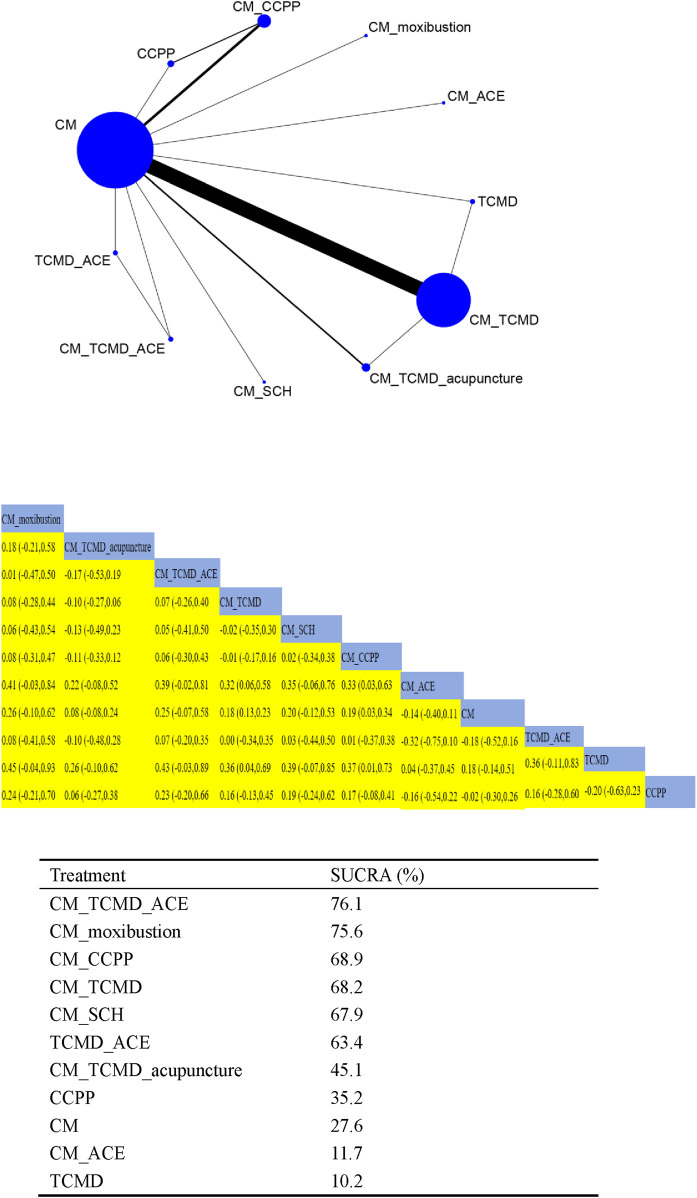
Schirmer trial network diagram, league diagram, SUCRA table.

A total of 41 studies reported effective rate as an outcome measure, involving 3,309 patients and 11 intervention strategies. Figure 3 presents the direct comparison network plot for effective rate. Nodes represent RCT intervention conditions, with node size proportional to the number of patients included. Lines represent direct comparisons between interventions, and line thickness reflects the number of RCTs contributing to each comparison.

Four closed-loop structures were identified within the network. The comparison between CM and CM + TCMD was the most prominent, with the thickest connecting line and the largest sample size. The league table demonstrated that all pairwise comparisons between interventions were statistically significant. According to the SUCRA values, the three most effective interventions were CM + TCMD + ACE (SUCRA = 76.1%), CM + moxibustion (SUCRA = 75.6%), and CM + CCPP(SUCRA = 68.9%). The effective rate of CM + TCMD + ACE was superior to that of CM + moxibustion [RR = 0.01,95CrI= (−0.47,0.50)]. The network geometry, league table, and SUCRA rankings are detailed in Figure 3.

### 3.4 Evidence network and network meta-analysis of core clinical manifestations

**FIGURE 4:**
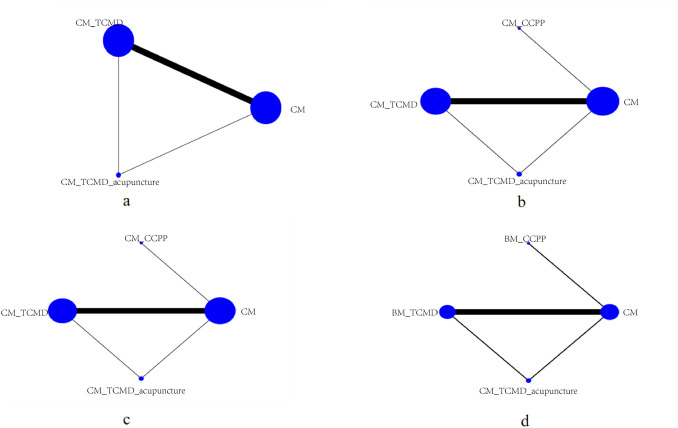
Schirmer trial network diagram [**(a)** diarrhea; **(b)** abdominal pain; **(c)** hematochezia; **(d)** tenesmus].

**FIGURE 5:**
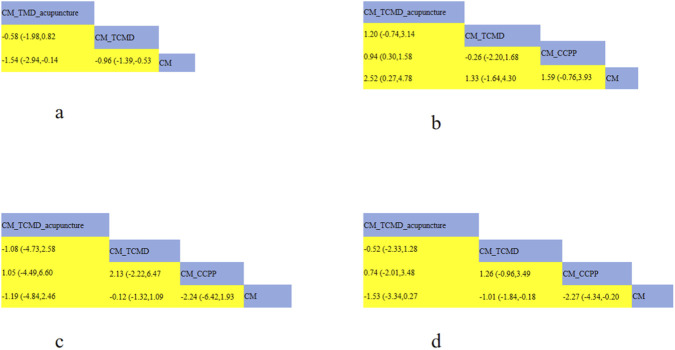
League diagram [**(a)** diarrhea; **(b)** abdominal pain; **(c)** hematochezia; **(d)** tenesmus].

**FIGURE 6:**
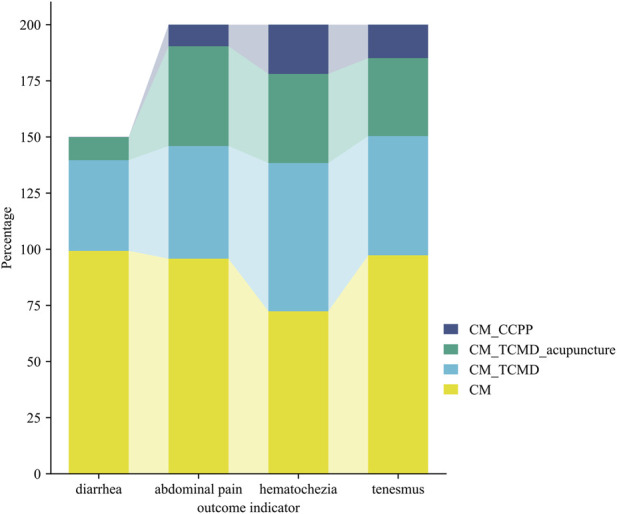
SUCRA diagram. The graph was generated online using the Microbioinfo platform (https://www.bioinformatics.com.cn/) with self-submitted raw data (Tang et al., 2023).

The common clinical manifestations of UC include diarrhea, abdominal pain, and mucopurulent and bloody stools (Wangchuk et al., 2024). In addition, other symptoms such as sallow or dull complexion, cold limbs, and a burning sensation in the anus may also be present. According to the inclusion and statistical criteria, a total of 15 studies reported diarrhea (1,102 patients), 13 studies reported abdominal pain (900 patients), 13 studies reported hematochezia (952 patients), and 7 studies reported tenesmus (544 patients). Heterogeneity was low for all these outcomes: abdominal pain (I^2^ = 9%), diarrhea (I^2^ = 2%), hematochezia (I^2^ = 4%), and tenesmus (I^2^ = 8%). Notably, diarrhea involved three interventions (CM,CM + TCMD,CM + TCMD + Acupuncture), whereas abdominal pain, hematochezia, and tenesmus all involved the same four interventions (CM,CM + TCMD,CM + TCMD + Acupuncture,CM + CCPP). All network meta-analyses contained one closed-loop, indicating that both direct and indirect evidence were available for these comparisons. Among these outcomes, the number of studies involving CM and CM + TCMD is the largest (Figure 4). According to the SUCRA scores (Figure 6), CM was superior to CM + TCMD in the outcomes of diarrhea and tenesmus {diarrhea [SMD = −0.96, 95%CrI (−1.39, −0.53), p < 0.05]; tenesmus [SMD = −1.01, 95%CrI (−1.84, −0.18), p < 0.05]} (Figure 5). However, for abdominal pain and hematochezia, no statistically significant differences were observed between CM and CM + TCMD (p > 0.05).

As shown in Figure 6, the ranking of SUCRA is as follows. Diarrhea: CM > CM + TCMD > CM + TCMD + acupuncture; abdominal pain: CM > CM + TCMD > CM + TCMD + acupuncture > CM + CCPP; hematochezia: CM > CM + TCMD > CM + TCMD + acupuncture > CM + CCPP; tenesmus: CM > CM + TCMD > CM + TCMD + acupuncture > CM + CCPP.

### 3.5 Evidence network and network meta-analysis of CRP

**FIGURE 7:**
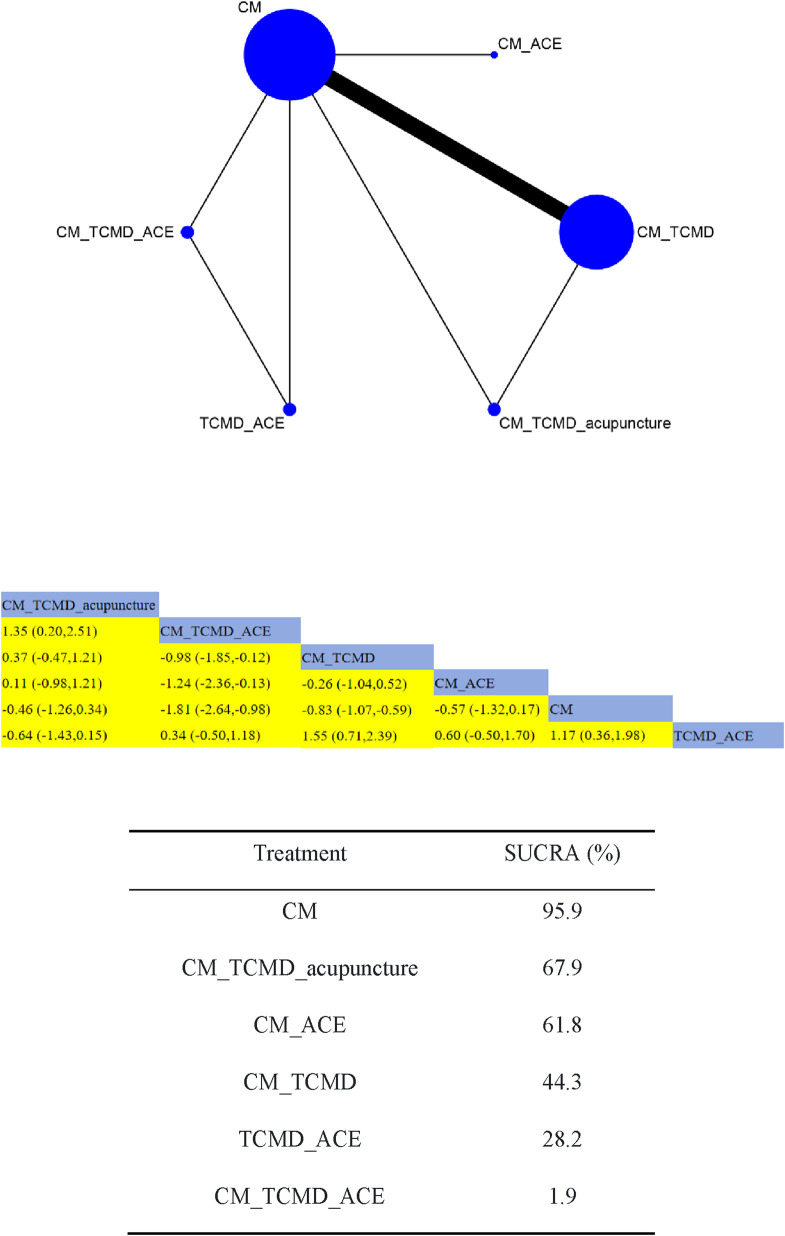
Schirmer trial network diagram, league diagram, SUCRA table.

Given the invasiveness of endoscopic examinations, and because C-reactive protein (CRP) is a commonly used indicator in the clinical assessment of patients with active UC, this outcome was also included in the present analysis (Sakurai and Saruta, 2023). Heterogeneity for CRP was extremely low (I^2^ = 0.1%). In this study fifteen RCTs reported data on CRP, involving 1,130 patients and six intervention strategies (CM, CM + ACE, CM + TCMD, CM + TCMD + Acupuncture, TCMD + ACE, CM + TCMD + ACE). Two closed loops were identified. The evidence network illustrating the differences in effectiveness among interventions is shown in Figure 7. The sample size was mainly concentrated in the CM group and the CM + TCMD group. CM was superior to CM + TCMD [SMD = −0.83, 95%CrI (−1.07, −0.59), p < 0.05], and CM + TCMD + ACE [SMD = −1.81 95%CrI (−2.64, −0.98), p < 0.05]. There were no statistically significant differences in CRP reduction between CM and the other intervention groups (CM + ACE, CM + TCMD + acupuncture, CM + ACE) (p > 0.05). The top three most effective interventions were CM (SUCRA = 95.9%), CM + TCMD + acupuncture (SUCRA = 67.9%), CM + ACE (SUCRA = 61.8%).

### 3.6 Evidence network and network meta-analysis of ESR

**FIGURE 8:**
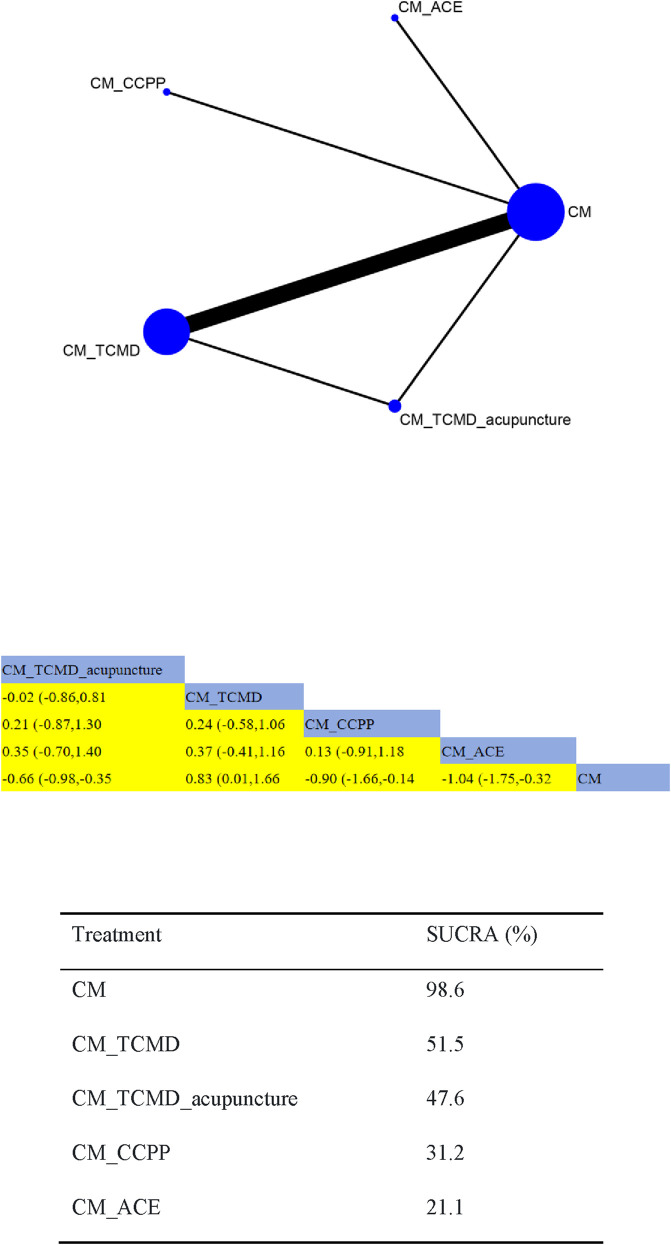
Schirmer trial network diagram, league diagram, SUCRA table.

Erythrocyte Sedimentation Rate (ESR) is an important inflammatory marker in patients with UC. According to the treatment target strategy proposed by the International Organization for the Study of Inflammatory Bowel Diseases (IOIBD), in addition to endoscopic healing and key indicators such as CRP, assessment of ESR is also recommended (Turner et al., 2021). Heterogeneity for ESR was low (I^2^ = 3%). A total of nine RCTs reported ESR as an outcome, involving 630 patients and five intervention strategies. As shown in Figure 8, the sample size was mainly concentrated in the CM group and the CM + TCMD group. In addition, a closed loop was formed among CM, CM + TCMD, and CM + TCMD + acupuncture. CM was superior to CM + TCMD [SMD = 0.83, 95%CrI (0.01,1.66)], CM + TCMD + acupuncture [SMD = −0.66, 95%CrI (−0.98, −0.35)], CM + CCPP [-0.90, 95%CrI (−1.66, −0.14)] and CM + ACE [-1.04, 95%CrI (−1.75, −0.32)] (p < 0.05). The three most effective interventions were CM (SUCRA = 98.6%), CM + TCMD(SUCRA = 51.5%), CM + TCMD + acupuncture (SUCRA = 47.6%).

### 3.7 Evidence network and network meta-analysis of cytokines

**FIGURE 9:**
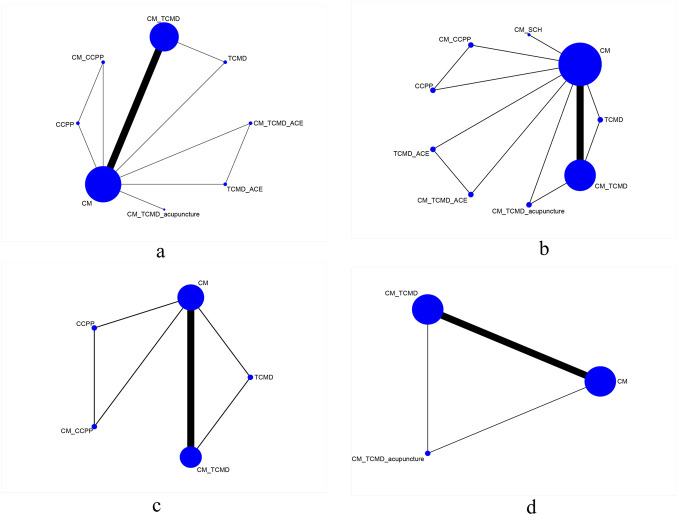
Schirmer trial network diagram [**(a)** TNF-α; **(b)** IL-6; **(c)** IL-8; **(d)** IL-10].

**FIGURE 10:**
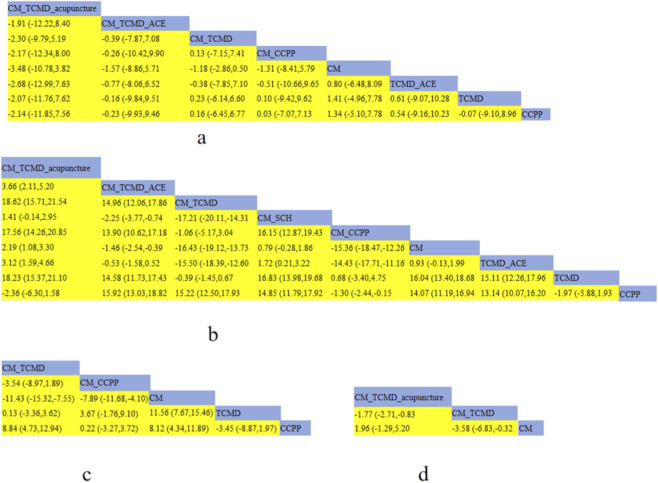
League diagram [**(a)** TNF-α; **(b)** IL-6; **(c)** IL-8; **(d)** IL-10].

Tumor necrosis factor-α (TNF-α), interleukin-6 (IL-6), and interleukin-8 (IL-8) are typical pro-inflammatory cytokines. During the progression of UC, TNF-α exerts pro-inflammatory effects, including activation of T cells and macrophages, as well as damage to epithelial cells. IL-8 can recruit neutrophils to sites of intestinal inflammation in UC (Nakase, Sato, Mizuno and Ikawa, 2022). A retrospective study involving 130 patients with UC showed that IL-6 may serve as a potential biomarker for evaluating disease activity in Chinese patients (Feng, Zhu, Liu, Xu and Shen, 2023). Interleukin-10 (IL-10) is a typical anti-inflammatory cytokine. In this meta-analysis, data on the following cytokines were reported across the included studies: 22 studies (1,902 patients) for TNF-α, 9 studies (829 patients) for IL-8, 14 studies (1,140 patients) for IL-6, and 12 studies (1,089 patients) for IL-10. Heterogeneity was low for all three interleukin outcomes: IL-6 (I^2^ = 4%), IL-8 (I^2^ = 6%), and IL-10 (I^2^ = 12%). The study samples were mainly concentrated in the CM group and the CM + TCMD group as shown in Figures 9, 10. The analysis revealed that the CM group significantly reduced IL-8 levels and was superior to the CM + TCMD group [SMD = −11.43, 95%CrI (−15.32, −7.55)], CM + CCPP group [SMD = −7.89, 95%CrI (−11.68, −4.10)], TCMD group [SMD = 11.56, 95%CrI (7.67,15.46)], and CCPP group [SMD = 8.12, 95%CrI (4.34, 11.89)] (P < 0.05). The CM + TCMD + acupuncture group showed a greater effect in reducing IL-6 levels than the CM + TCMD group [SMD = 18.62, 95%CrI (15.71, 21.54)] and the CM alone group [SMD = 2.19, 95%CrI (1.08, 3.30)] (P < 0.05). In the analysis of IL-10 related outcomes, three intervention strategies were included. Among them, the CM + TCMD group exhibited a significantly better regulatory effect on IL-10 than the CM monotherapy group [SMD = −3.58, 95%CrI (−6.83, −0.32)] and the CM + TCMD + acupuncture group [SMD = −1.77, 95%CrI (−2.71, −0.83)] (P < 0.05). Although six intervention strategies were included in the network meta-analysis of TNF-α, and the number of enrolled patients for this outcome was the largest among all inflammatory indicators, no statistically significant differences were observed among these six interventions with respect to TNF-α regulation.

**FIGURE 11:**
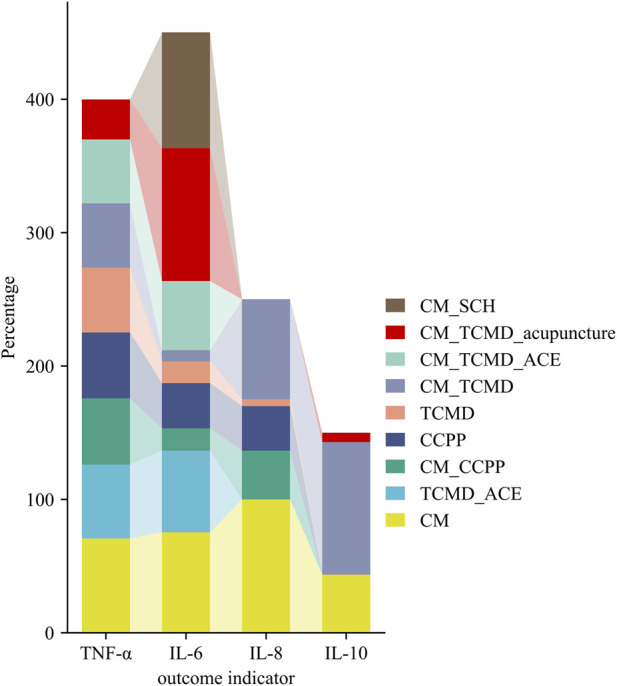
SUCRA diagram. The graph was generated online using the Microbioinfo platform (https://www.bioinformatics.com.cn/) with self-submitted raw data (Tang et al., 2023).

As shown in Figure 11, the ranking of SUCRA is as follows. IL-6: CM + TCMD + acupuncture > CM + SCH > CM > TCMD + ACE > CM + TCMD + ACE > CCPP > CM + CCPP > TCMD > CM + TCMD; IL-8: CM > CM + TCMD > CM + CCPP > CCPP > TCMD; IL-10: CM + TCMD > CM > CM + TCMD + acupuncture.

### 3.8 Adverse reactions

A total of Thirteen randomized controlled trials reported adverse events, three of the included studies reported recurrence rates at 6 months and 12 months follow-up. The reported adverse events included intestinal stricture, rash, fatigue, nausea and vomiting, among others. Thirteen studies did not implement any intervention for adverse events. Details are presented in Supplementary Table S3.

### 3.9 Consistency analysis

Bayesian P values generated by the node-splitting method were used to assess consistency between direct and indirect comparisons. The results showed that, among all outcome indicators included in this study, the effective rate and traditional Chinese medicine–related clinical manifestations demonstrated good consistency (P > 0.05); therefore, a consistency model was adopted for the network meta-analysis of these outcomes. In contrast, for indicators showing poor consistency (such as inflammatory biomarkers), an inconsistency model was applied in the network meta-analysis.

### 3.10 Publication bias

**FIGURE 12:**
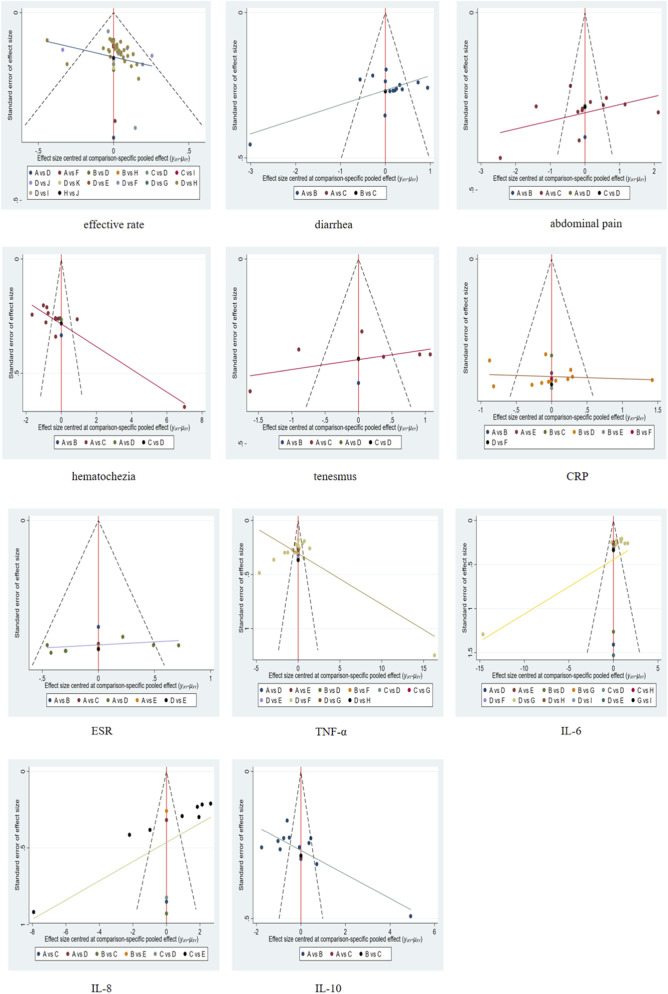
Funnel plot of outcome indicator.

The comparative-corrected funnel plot revealed that the majority of included studies were symmetrically distributed around the zero line. However, a small proportion of studies exhibited a scattered distribution pattern. This finding suggests the possible presence of publication bias as well as small-study effects (Figure 12).

### 3.11 Sensitivity analysis

Considering that potent drugs in the control group of the included studies may introduce heterogeneity, we adopted a sensitivity analysis method of stratified exclusion to verify whether the inclusion of potent drugs (such as biological agents and systemic hormones) dominates the study results. Re-conduct the network meta-analysis in accordance with the analytical methods of the original study, generate new effect sizes and the league table of intervention measures, and compare the differences in effect sizes before and after exclusion. In this study, the relevant outcome indicators involving infliximab include response rate, diarrhea, abdominal pain, hematochezia, TNF-α, IL-10, and IL-6. After excluding the studies involving infliximab, except for a slight fluctuation in the analysis results of abdominal pain indicators, the change trends of the other indicators remained consistent, with a large overlap range of effect sizes and no change in statistical significance. This indicates that the research results have good stability. Meanwhile, the outcome indicators related to systemic hormones in this study include response rate, abdominal pain, and tenesmus. After excluding the studies involving systemic hormones, the results of the response rate indicator showed good stability; however, for the two indicators of abdominal pain and tenesmus, since the corresponding intervention measures were removed along with the exclusion of the studies, sensitivity analysis could not be conducted.

## 4 Discussion

Ulcerative colitis (UC) is a disabling, lifelong inflammatory bowel disease (IBD), with its global prevalence increasing steadily over recent years (Jairath, Narula, Ungaro, Romo Bautista and Adsul, 2025). Within the theoretical framework of TCM, there is no modern disease entity directly corresponding to UC. However, through long-term observation and systematic summarization of clinical manifestations, disease course characteristics, and pathogenic evolution, its core syndrome is considered to closely correspond to the category of “Jiuli” (chronic dysentery) in TCM. TCM has a history of several thousand years in the treatment of Jiuli. Physicians of successive dynasties accumulated extensive clinical experience through long-term practice, providing a solid foundation for the inheritance and development of TCM-based therapeutic strategies for this condition. Accordingly, this network meta-analysis was conducted to explore and compare the efficacy of integrated TCM–CM interventions in the treatment of UC.

A total of 46 RCTs were included in this study. CM treatments mainly included mesalazine and sulfasalazine, while the TCM interventions covered decoctions, acupuncture, commercial Chinese polyherbal preparation, and single-herb therapies, among others. Ultimately, 11 outcome indicators were included, which were mainly categorized into three types: effective rate, clinical manifestations (abdominal pain, diarrhea, hematochezia, and tenesmus), and inflammatory indicators (CRP, ESR, TNF-α, IL-6, IL-8, and IL-10). Both direct and indirect comparisons between different intervention measures were conducted, covering both single-use and combined-use treatment strategies. The results showed that the combined intervention CM + TCMD + ACE ranked highly (76.1%) and demonstrated a prominent advantage in terms of total treatment effective rate. In terms of improvement of core clinical symptoms, CM monotherapy ranked highest across all evaluated outcomes, including diarrhea (99.3%), abdominal pain (95.8%), hematochezia (72.4%), and tenesmus (97.3%). Furthermore, the single use of CM also ranked highly in the regulation of inflammatory indicators such as CRP, ESR, and IL-8. However, for the IL-6 outcome indicator, the combined intervention CM + TCMD + acupuncture ranked highest (99.5%). A network pharmacology study showed that quercetin, kaempferol, ginsenoside Rh2, and other components contained in Wumei Pills are key active ingredients in the treatment of UC (Huang et al., 2021). These active components possess anti-inflammatory and antioxidant properties and can regulate immune responses at the molecular level. Another study indicated that Baitouweng Decoction can alleviate symptoms of experimental UC in mice by regulating the intestinal microbiota and the IL-6/STAT3 signaling pathway (Xuan-Qing et al., 2021).

In our research, six studies involving 288 UC patients used Wumei Pills as an intervention, while seven studies, including a total of 239 UC patients, involved intervention with Baitouweng Decoction. The results also showed that intervention combinations containing Chinese herbal decoctions had significant advantages in reducing IL-6 levels and increasing IL-10 levels. In addition, the combined intervention CM + TCMD (99.3%) demonstrated the best efficacy in regulating IL-10. Among the included studies, several indicators closely related to the occurrence and progression of UC were also reported (Geremia et al., 2014; Guo et al., 2020), such as interleukin-4 (IL-4), interleukin-17 (IL-17), interleukin-1β (IL-1β), intestinal flora, and immunoglobulins. However, network meta-analysis was not performed for these outcomes due to an insufficient number of eligible studies. This study focuses on integrated traditional Chinese and conventional medcine (TCM-CM) treatment for UC. It includes not only classic conventional medcine (such as mesalazine and sulfasalazine) but also diverse TCM interventions, including decoctions, acupuncture, and proprietary TCM medicines. This study provides quantitative evidence on the differences in efficacy among integrated TCM-CM treatment regimens for UC and helps fill the gap in multi-regimen comparisons in this field. Regrettably, the number of included studies reporting adverse reactions was extremely limited, which precluded a clear and comprehensive assessment of the safety profile of integrated TCM-CM treatment. In addition, due to the limited adoption of TCM in countries outside China, all participants included in this study were Chinese patients. This may introduce regional, linguistic, and ethnic biases into the study findings. Furthermore, most of the included studies did not fully describe the details of random sequence generation, allocation concealment, and blinding implementation, which may lead to selection bias and performance bias, and exert a certain potential impact on the authenticity and reliability of the outcome indicators. At the same time, this study also has methodological limitations that should be objectively noted in the context of its design and analysis. First of all, to adapt to the characteristic of this study that the included research covers a wide time span (including studies published in different periods), we adopted the Cochrane Risk of Bias Assessment Tool (Version 1 of the Cochrane Risk of Bias Assessment Tool) to conduct bias risk assessment on the included studies, so as to ensure the reliability of the research conclusions. The sensitivity analysis was limited, as it did not explore the effects of different exclusion orders or drug dose stratifications on results. Additionally, due to heterogeneity among included studies, even maximal evaluation via stratified exclusion could not fully eliminate potential biases from differences in drug mechanisms or study designs, which requires further optimization in future research. Therefore, caution is warranted when interpreting and generalizing the conclusions. Accordingly, we recommend that future large-scale randomized controlled trials involving TCM be conducted in other countries to further enhance the reliability and scientific validity of the evidence and to provide broader support for the application of TCM in the treatment of UC.

## 5 Conclusion

Based on the above findings, the selection of clinical intervention regimens for ulcerative colitis should follow the principle of individualized treatment according to therapeutic goals. Monotherapy with conventional medicine (CM) is preferable for rapidly relieving symptoms (e.g., diarrhea, abdominal pain) and reducing routine inflammatory markers (CRP, ESR), given its definite efficacy and high evidence consistency. For improving the overall clinical response rate or regulating specific cytokines (IL-6, IL-10), integrated regimens of CM combined with Traditional Chinese Medicine Decoction (TCMD), with or without acupuncture, are more clinically valuable, and can be flexibly selected based on patients’ acceptance of acupuncture and individual conditions.

Notably, the current study is limited by the lack of valid clinical activity scores and endoscopic mucosal healing data—essential endpoints required by international regulatory authorities (FDA, EMA) for confirming definitive therapeutic efficacy. Thus, although TCM-integrated regimens show benefits in symptom relief and biomarker regulation, further high-quality clinical trials incorporating endoscopic and standardized clinical scoring are needed to verify their definite clinical efficacy.

## Data Availability

The original contributions presented in the study are included in the article/Supplementary Material, further inquiries can be directed to the corresponding author.
