# Supplementary material for: Systematic review and network meta-analysis of integrated traditional Chinese and conventional medicine for ulcerative colitis
Source: Front Pharmacol. 2026 Jun 25;17:1785134. doi: 10.3389/fphar.2026.1785134 (PMC13345878; doi:10.3389/fphar.2026.1785134)

*Supplementary Material*

1. **Retrieval strategy**

**1.1 retrieval strategy for Chinese databases**

((((((Abstract=integrative Chinese and Western medicine OR Abstract=integrative Chinese-Western medicine therapy OR Abstract=integrative Chinese-Western medicine treatment OR Abstract=combination of Chinese materia medica and Western medicine OR Abstract=combination of traditional Chinese medicine and Western medicine OR Abstract=combination of Chinese materia medica and Western medicine OR Abstract=combination of traditional Chinese medicine and Western medicine) OR Abstract=integrative medicine) OR (Abstract=combination of Chinese traditional AND Abstract=western medicine)) OR (Abstract=integrated traditional Chinese AND Abstract=western medicine)) OR (Abstract=integration of traditional AND

Abstract=western medicine)) OR Abstract=integrative medicine) AND ((((Abstract=ulcerative colitis OR Abstract=nonspecific ulcerative colitis) OR Abstract=ulcerative colitis) OR Abstract=ulcerative colonitis) OR Abstract=ulcerous colonitis)) AND ((((Abstract=random OR Abstract=random grouping) OR Abstract=randomized controlled) OR Abstract=randomized controlled trial) OR

Abstract=randomized controlled study))

**1.2 retrieval strategy for English databases**

((Ulcerative Colitis[MeSH Terms]) OR (Colitis Gravis[Title/Abstract] OR Idiopathic Proctocolitis[Title/Abstract] OR Ulcerative Colitis chronic ulcerative

colitis[Title/Abstract] OR colitis ulcerative[Title/Abstract] OR colitis

ulcerosa[Title/Abstract] OR colitis ulcerosa chronica[Title/Abstract] OR histiocytic

ulcerative colitis[Title/Abstract] OR mucosal colitis[Title/Abstract] OR ulcerative

colorectitis[Title/Abstract] OR ulcerative procto colitis[Title/Abstract] OR ulcerative proctocolitis[Title/Abstract] OR ulcerous colitis[Title/Abstract] OR ulcerative

colitis[Title/Abstract])) AND ((((traditional Chinese medicine[MeSH Terms]) OR

(western medicine[MeSH Terms])) OR (Integrative medicine[MeSH Terms])) OR

(Integrative medicine[Title/Abstract] OR western medicine[Title/Abstract] OR Zhong Yi Xue[Title/Abstract] OR Chung I Hsueh[Title/Abstract] OR Chinese Traditional

Medicine[Title/Abstract] OR Traditional Chinese Medicine[Title/Abstract] OR

Traditional Tongue Diagnosis[Title/Abstract] OR Traditional Tongue

Diagnoses[Title/Abstract] OR Traditional Tongue Assessment[Title/Abstract] OR

Traditional Tongue Assessments[Title/Abstract] OR Chinese medicine[Title/Abstract] OR Chinese herbal medicine[Title/Abstract] OR Chinese traditional

medicine[Title/Abstract]))

1. **Supplementary Table S1 Components of traditional Chinese medicine decoction**

Table S1

| Chinese herbal formula | Components | |  |
| --- | --- | --- | --- |
|  | Herb name | Botanical origin (Latin name) | Family/Medicinal part |
| Baitouweng Decoction | 1.Baitouweng  2.Huanglian  3.Huangbai  4.Qinpi | *1.Pulsatilla chinensis (Bunge) Regel*  *2.Coptis chinensis Franch., Coptis deltoidea C. Y. Cheng et Hsiao, or Coptis teeta Wall.*  *3.Phellodendron chinense Schneid.*  *4.Fraxinus rhynchophylla Hance, Fraxinus chinensis Roxb., Fraxinus szaboana Lingelsh., or Fraxinus stylosa Lingelsh.* | 1.Ranunculaceae; Dried root  2.Ranunculaceae; Dried rhizome  3.Rutaceae; Dried bark  4.Oleaceae; Dried branch bark or stem bark |
| Gegenqinlian Decoction | 1.Gegen  2.Huangqin  3.Huanglian  4.Gancao | *1.Pueraria lobata (Willd.) Ohwi*  *2.Scutellaria baicalensis Georgi*  *3.Coptis chinensis Franch., Coptis deltoidea C. Y. Cheng et Hsiao, or Coptis teeta Wall.*  *4.Glycyrrhiza uralensis Fisch., Glycyrrhiza inflata Batal. or Glycyrrhiza glabra L* | 1.Fabaceae; Dried root  2.Lamiaceae; Dried root  3.Rutaceae; Dried bark  4.Fabaceae; Dried roots and rhizomes |
| Clam Shell Powder | 1.Walengzi  2.Muxiang  3.Taoren  4.Dongguazi  5.Baitouweng  6.Huanglian  7.Huangbai  8.Qinpi | *1.Arca subcrenata Lischke, Arca granosa Linnaeus, or Arca inflata Reeve*  *2.Aucklandia lappa Decne.*  *3.Prunus persica (L.) Batsch Or Prunus davidiana (Carr.) Franch.*  *4.Benincasa hispida (Thunb.) Cogn.*  *5.Pulsatilla chinensis (Bunge) Regel*  *6.Coptis chinensis Franch., Coptis deltoidea C. Y. Cheng et Hsiao, or Coptis teeta Wall.*  *7.Phellodendron chinense Schneid.*  *8.Fraxinus rhynchophylla Hance, Fraxinus chinensis Roxb., Fraxinus szaboana Lingelsh., or Fraxinus stylosa Lingelsh.* | 1.Arcidae; Dried shell  2.Asteraceae; Dried root  3.Rosaceae; Dried ripe seed  4.Cucurbitaceae; Dried ripe seed  5.Ranunculaceae; Dried root  6.Rutaceae; Dried bark  7.Rutaceae; Dried bark  8.Oleaceae; Dried branch bark or stem bark |
| Gancao Xiexin Decoction | 1.Gancao  2.Huangqin  3.Huanglian  4.Banxia  5.Dangshen  6.Paojiang  7.Dazao | 1.*Glycyrrhiza uralensis Fisch., Glycyrrhiza inflata Batal. or Glycyrrhiza glabra L*  *2.Scutellaria baicalensis Georgi*  *3.Coptis chinensis Franch., Coptis deltoidea C. Y. Cheng et Hsiao, or Coptis teeta Wall.*  *4.Pinellia ternata (Thunb.) Breit.*  *5.Codonopsis pilosula (Franch.) Nannf.*  *6.Zingiber officinale Rosc.*  *7.Ziziphus jujuba Mill.* | 1.Fabaceae; Dried roots and rhizomes  2.Lamiaceae; Dried root  3.Rutaceae; Dried bark  4.Araceae; Dried tuber  5. Campanulaceae; Dried root  6. Zingiberaceae; Processed dried rhizome  7. Rhamnaceae; Dried ripe fruit |
| Huangqin Decoction | 1.Huangqin  2.Baishao  3.Dazao  4.Gancao | *1.Scutellaria baicalensis Georgi*  *2.Paeonia lactiflora Pall.*  *3.Ziziphus jujuba Mill.*  *4.Glycyrrhiza uralensis Fisch., Glycyrrhiza inflata Batal. or Glycyrrhiza glabra L* | 1.Lamiaceae; Dried root  2.Ranunculaceae; Dried root  3.Ranunculaceae; Dried root  4. Fabaceae; Dried roots and rhizomes |

Table S1 (cont.)

| Chinese herbal formula | Components | |  |
| --- | --- | --- | --- |
|  | Herb name | Botanical origin (Latin name) | Family/Medicinal part |
| Wumei Pill | 1.Wumei  2.Huajiao  3.Xixin  4.Huanglian  5.Huangbai  6.Ganjiang  7.Fizi(zhi)  8.Guizhi  9.Renshen  10.Danggui | *1.Prunus mume (Siebold et Zucc.) Siebold et Zucc.*  *2.Zanthoxylum bungeanum Maxim.*  *3.Asarum heterotropoides Fr. Schmidt var. mandshuricum (Maxim.) Kitag.*  *or Asarum sieboldii Miq. var. seoulense (Nakai) C. Y. Cheng et C. S. Yang*  *4.Coptis chinensis Franch., Coptis deltoidea C. Y. Cheng et Hsiao, or Coptis teeta Wall.*  *5.Phellodendron chinense Schneid. or Phellodendron amurense Rupr.*  *6.Zingiber officinale Rosc.*  *7.Aconitum carmichaelii Debeaux*  *8.Cinnamomum cassia Presl*  *9.Panax ginseng C. A. Mey.*  *10.Angelica sinensis (Oliv.) Diels* | 1.Rosaceae; Dried nearly ripe fruit  2.Rutaceae; Dried ripe pericarp  3. Aristolochiaceae; Dried root and rhizome  4. Rutaceae; Dried bark  5. Rutaceae; Dried bark  6.Zingiberaceae; Dried rhizome  7.Ranunculaceae; Dried daughter root (processed)  8.Lauraceae; Dried twig  9.Araliaceae; Dried root and rhizome  10.Apiaceae; Dried root |
| Huanglian Jiedu Decoction | 1.Huanglian  2.Huangqin  3.Huangbai  4.Zhizi | *1.Coptis chinensis Franch., Coptis deltoidea C. Y. Cheng et Hsiao, or Coptis teeta Wall.*  *2.Scutellaria baicalensis Georgi*  *3.Phellodendron chinense Schneid. or Phellodendron amurense Rupr.*  *4.Gardenia jasminoides Ellis* | 1.Ranunculaceae; Dried rhizome  2.Lamiaceae; Dried root  3.Rutaceae; Dried bark  4.Rubiaceae; Dried ripe fruit |
| Shaoyao Decoction | 1.Danggui  2.Huanglian  3.Baishao  4.Binglang  5.Muxiang  6.Gancao  7.Dahuang  8.Huangqin  9.Rougui | *1.Angelica sinensis (Oliv.) Diels*  *2.Coptis chinensis Franch., Coptis deltoidea C.Y. Cheng et Hsiao, or Coptis teeta Wall.*  *3.Paeonia lactiflora Pall.*  *4.Areca catechu L.*  *5.Aucklandia lappa Decne.*  *6.Glycyrrhiza uralensis Fisch., Glycyrrhiza inflata Batal., or Glycyrrhiza glabra L.*  *7.Rheum palmatum L., Rheum tanguticum Maxim. ex Balf., or Rheum officinale Baill.*  *8.Scutellaria baicalensis Georgi*  *9.Cinnamomum cassia Presl* | 1.Apiaceae; Dried root  2.Ranunculaceae; Dried rhizome  3.Ranunculaceae; Dried root  4.Arecaceae; Dried ripe seed  5.Asteraceae; Dried root  6.Fabaceae; Dried roots and rhizomes  7.Polygonaceae; Dried root and rhizome  8.Lamiaceae; Dried root  9.Lauraceae; Dried bark |
| Buzhong Yiqi Decoction | 1.Huangqi  2.Gancao  3.Renshen  4.Danggui  5.Chenpi  6.Shengma  7.Chaihu  8.Baizhu | *1.Astragalus membranaceus (Fisch.) Bunge var. mongholicus (Bunge) Hsiao*  *2.Glycyrrhiza uralensis Fisch., Glycyrrhiza inflata Batal., or Glycyrrhiza glabra L.*  *3.Panax ginseng C. A. Mey.*  *4.Angelica sinensis (Oliv.) Diels*  *5.Citrus reticulata Blanco*  *6.Cimicifuga foetida L., Cimicifuga heracleifolia Kom., or Cimicifuga dahurica (Turcz.) Maxim.*  *7.Bupleurum chinense DC. or Bupleurum scorzonerifolium Willd.*  *8.Atractylodes macrocephala Koidz.* | 1.Fabaceae; Dried root  2.Fabaceae; Dried roots and rhizomes (processed)  3.Araliaceae; Dried root and rhizome  4.Apiaceae; Dried root  5.Rutaceae; Dried ripe pericarp  6.Ranunculaceae; Dried rhizome  7.Apiaceae; Dried root  8.Asteraceae; Dried rhizome |

Table S1 (cont.)

| Chinese herbal formula | Components | |  |
| --- | --- | --- | --- |
|  | Herb name | Botanical origin (Latin name) | Family/Medicinal part |
| Fuzilizhong Decoction | 1.Fuzi(zhi)  2.Renshen  3.Ganjiang  4.Gancao(zhi)  5.Baizhu | *1.Aconitum carmichaelii Debeaux*  *2.Panax ginseng C. A. Mey.*  *3.Zingiber officinale Rosc.*  *4.Glycyrrhiza uralensis Fisch., Glycyrrhiza inflata Batal., or Glycyrrhiza glabra L.*  *5.Atractylodes macrocephala Koidz.* | 1.Ranunculaceae; Dried daughter root (processed)  2.Araliaceae; Dried root and rhizome  3.Zingiberaceae; Dried rhizome  4.Fabaceae; Dried roots and rhizomes (processed)  5.Asteraceae; Dried rhizome |
| Sishen pill | 1.Roudoukou  2.Buguzhi  3.Wuweizi  4.Wuzhuyu  5.Dazao | *1.Myristica fragrans Houtt.*  *2.Psoralea corylifolia L.*  *3.Schisandra chinensis (Turcz.) Baill.*  *4.Evodia rutaecarpa (Juss.) Benth.*  *5.Ziziphus jujuba Mill.* | 1.Myristicaceae; Dried aril and seed  2.Fabaceae; Dried ripe fruit  3.Schisandraceae; Dried ripe fruit  4.Rutaceae; Dried nearly ripe fruit  5.Rhamnaceae; Dried ripe fruit |
| Jingjie Lianqiao Decoction | 1.Jingjie  2.Lianqiao  3.Fangfeng  4.Danggui  5.Chuanxiong  6.Baishao  7.Chaihu  8.Zhike  9.Huangqin  10.Zhizi  11.Baizhi  12.Jiegen  13.Gancao(zhi) | *1.Schizonepeta tenuifolia Briq.*  *2.Forsythia suspensa (Thunb.) Vahl*  *3.Saposhnikovia divaricata (Turcz.) Schischk.*  *4.Angelica sinensis (Oliv.) Diels*  *5.Ligusticum chuanxiong Hort.*  *6.Paeonia lactiflora Pall.*  *7.Bupleurum chinense DC.*  *8.Citrus aurantium L.*  *9.Scutellaria baicalensis Georgi*  *10.Gardenia jasminoides Ellis*  *11.Angelica dahurica (Fisch. ex Hoffm.) Benth. et Hook. f.*  *12.Platycodon grandiflorus (Jacq.) A. DC.*  *13.Glycyrrhiza uralensis Fisch.* | 1.Lamiaceae; Dried aerial parts  2.Oleaceae; Dried fruit  3.Apiaceae; Dried root  4.Apiaceae; Dried root  5.Apiaceae; Dried rhizome  6.Paeoniaceae; Dried root  7.Apiaceae; Dried root  8.Rutaceae; Dried unripe fruit  9.Lamiaceae; Dried root  10.Rubiaceae; Dried ripe fruit  11.Apiaceae; Dried root  12.Campanulaceae; Dried root  13.Fabaceae; Dried root and rhizome |
| Houpu Xialing Decoction | 1.Huoxiang  2.Houpu  3.Banxia  4.Fuling  5.Kuxingren  6.Yiyiren  7.Baidoukou  8.Zhuliing  9.Zexie  10.Dandouchi  11.Tongcao | *1.Agastache rugosa (Fisch. et Mey.) O. Ktze.*  *2.Magnolia officinalis Rehd. et Wils.*  *3.Pinellia ternata (Thunb.) Breit.*  *4.Poria cocos (Schw.) Wolf*  *5.Prunus armeniaca L. var. ansu Maxim.*  *6.Coix lacryma-jobi L. var. ma-yuen (Roman.) Stapf*  *7.Amomum kravanh Pierre ex Gagnep.*  *8.Polyporus umbellatus (Pers.) Fries*  *9.Alisma orientale (Sam.) Juzep.*  *10.Glycine max (L.) Merr.*  *11.Tetrapanax papyriferus (Hook.) K. Koch* | 1.Lamiaceae; Dried aerial parts  2.Magnoliaceae; Dried bark, root bark and branch bark  3.Araceae; Dried tuber  4.Polyporaceae; Dried sclerotium  5.Rosaceae; Dried ripe seed  6.Poaceae; Dried ripe kernel  7.Zingiberaceae; Dried ripe seed  8.Polyporaceae; Dried sclerotium  9.Alismataceae; Dried tuber  10.Fabaceae; Fermented preparation of seed  11.Araliaceae; Dried stem pith |

Table S1 (cont.)

| Chinese herbal formula | Components | |  |
| --- | --- | --- | --- |
|  | Herb name | Botanical origin (Latin name) | Family/Medicinal part |
| Xiaoyao Powder | 1.Chaihu  2.Danggui  3.Baishao  4.Baizhu  5.Fuling  6Gancao(zhi)  7.Bohe  8.Shengjiang | *1.Bupleurum chinense DC. or Bupleurum scorzonerifolium Willd.*  *2.Angelica sinensis (Oliv.) Diels*  *3.Paeonia lactiflora Pall.*  *4.Atractylodes macrocephala Koidz.*  *5.Poria cocos (Schw.) Wolf*  *6.Glycyrrhiza uralensis Fisch., G. inflata Batal., or G. glabra L.*  *7.Mentha haplocalyx Briq.*  *8.Zingiber officinale Rosc.* | 1.Apiaceae; Dried root  2.Apiaceae; Dried root  3. Ranunculaceae; Dried root  4.Asteraceae; Dried rhizome  5.Polyporaceae; Dried sclerotium  6.Fabaceae; Dried root and rhizome (processed)  7.Lamiaceae; Dried aerial parts  8.Zingiberaceae; Fresh rhizome |
| Jianpi Zhixie Decoction | 1.Yiyiren  2.Dangshen  3.Baishao  4.Baibiandou  5.Baizhu  6.Baijiang  7.Danggui  8.Muxiang  9.Pugongying  10.Chishizhi  11.Sharen  12.Wumei  13.Gancao | *1.Coix lacryma-jobi L. var. ma-yuen (Roman.) Stapf*  *2. Codonopsis pilosula (Franch.) Nannf.*  *3.Paeonia lactiflora Pall.*  *4.Lablab purpureus (L.) Sweet*  *5.Atractylodes macrocephala Koidz.*  *6.Patrinia scabiosaefolia Fisch.*  *7.Angelica sinensis (Oliv.) Diels*  *8.Aucklandia lappa Decne.*  *9.Taraxacum mongolicum Hand.-Mazz.*  *10.Halloysitum Rubrum*  *11.Amomum villosum Lour.*  *12.Prunus mume (Sieb.) Sieb. et Zucc.*  *13.Glycyrrhiza uralensis Fisch., Glycyrrhiza inflata Bat., or Glycyrrhiza glabra L.* | 1.Poaceae; Dried ripe kernel  2.Campanulaceae; Dried root  3.Ranunculaceae; Dried root  4.Fabaceae; Dried ripe seed  5.Asteraceae; Dried rhizome  6.Caprifoliaceae; Dried whole herb  7.Apiaceae; Dried root  8.Asteraceae; Dried root  9.Asteraceae; Dried whole herb  10. Mainly contains [Al₄(Si₄O₁₀)(OH)₈・4H₂O]  11.Zingiberaceae; Dried ripe fruit  12.Rosaceae; Dried nearly ripe fruit  13.Fabaceae; Dried root and rhizome |
| Shenling Baizhu Powder | 1.Dangshen  2.Huangqi  3.Baizhu  4.Fuling  5.Chenpi  6.Fabanxia  7.Muxiang  8.Sharen  9.Yiyiren  10.Zhishi  11.Houpu  12.Gancao | *1.Codonopsis pilosula (Franch.) Nannf.*  *2.Astragalus membranaceus (Fisch.) Bunge var. mongholicus (Bunge) Hsiao*  *3.Atractylodes macrocephala Koidz.*  *4.Poria cocos (Schw.) Wolf*  *5.Citrus reticulata Blanco*  *6.Pinellia ternata (Thunb.) Breit.*  *7.Aucklandia lappa Decne.*  *8.Amomum villosum Lour.*  *9.Coix lacryma-jobi L. var. ma-yuen (Roman.) Stapf*  *10.Citrus aurantium L. or Citrus sinensis Osbeck*  *11.Magnolia officinalis Rehd. et Wils.*  *12.Glycyrrhiza uralensis Fisch., Glycyrrhiza inflata Bat., Glycyrrhiza glabra L.* | 1.Campanulaceae; Dried root  2.Fabaceae; Dried root  3.Asteraceae; Dried rhizome  4.Polyporaceae; Dried sclerotium  5.Rutaceae; Dried ripe pericarp  6.Araceae; Processed tuber  7.Asteraceae; Dried root  8.Zingiberaceae; Dried ripe fruit  9.Poaceae; Dried ripe kernel  10.Rutaceae; Dried young fruit  11.Magnoliaceae; Dried bark, root bark and branch bark  12.Fabaceae; Dried root and rhizome |
| Changshu Granules | 1.Chenpi  2.Fangfeng  3.Yiyiren  4.Baidoukou  5.Muxiang  6.Huanglian  7.Baizhu  8Baishao  9.Fuling  10.Zexie  11.Gancao | *1.Citrus reticulata Blanco*  *2.Saposhnikovia divaricata (Turcz.) Schischk.*  *3. Coix lacryma-jobi L. var. ma-yuen (Roman.) Stapf*  *4.Amomum kravanh Pierre ex Gagnep.*  *5.Aucklandia lappa Decne.*  *6.Coptis chinensis Franch., Coptis deltoidea C.Y. Cheng et Hsiao, or Coptis teeta Wall*  *7.Atractylodes macrocephala Koidz.*  *8.Paeonia lactiflora Pall.*  *9.Poria cocos (Schw.) Wolf*  *10.Alisma orientale (Sam.) Juzep. or Alisma plantago-aquatica Linn.*  *11.Glycyrrhiza uralensis Fisch., Glycyrrhiza inflata Bat., Glycyrrhiza glabra L.* | 1.Rutaceae; Dried ripe pericarp  2.Apiaceae; Dried root  3.Poaceae; Dried ripe kernel  4. Zingiberaceae; Dried ripe seed  5. Asteraceae; Dried root  6. Ranunculaceae; Dried rhizome  7.Asteraceae; Dried rhizome  8.Ranunculaceae; Dried root  9.Polyporaceae; Dried sclerotium  10.Alismataceae; Dried rhizome  11.Fabaceae; Dried root and rhizome |

Note: All information on traditional Chinese medicines in the table is obtained from *Pharmacopoeia of the People's Republic of China* (2020 Edition Volume I).

**3 Supplementary Table S2 commercial Chinese polyherbal preparation**

| Generic Name | Main Ingredients | National Drug Standard |
| --- | --- | --- |
| Bupi Yichang Wan | Huangqi.*Astragalus membranaceus (Fisch.) Bunge var. mongholicus (Bunge) Hsiao*  Dangshen.*Codonopsis pilosula (Franch.) Nannf.*  Baishao.*Paeonia lactiflora Pall.*  Danggui.*Angelica sinensis (Oliv.) Diels*  Baizhu.*Atractylodes macrocephala Koidz.*  Sharen.*Amomum villosum Lour, Amomum villosum Lour. var. xanthioides T. L. Wu et Senjen, Amomum longiligulare T. L. Wu*  Yanhusuo.*Corydalis yanhusuo W. T. Wang* | The product mainly complies with the National Drug Standard (Ministerial Standard for Traditional Chinese Medicine) with the standard number: WS₃-B-3418-98. |
| Xilei San | Xiangyaxie. *Odus Elephatis*  Rengongniuhuang (Artificial Calculus Bovis). *Prepared by mixing and processing bovine bile powder, cholic acid, hyodeoxycholic acid, taurine, bilirubin, cholesterol, trace elements, etc.*  Qiangdai.*Baphicacanthus cusia (Nees) Bremek, Polygonum tinctorium Ait, Isatis indigotica Fort.* | The current national pharmaceutical standard WS₃-B-0452-90-2016 (originally issued as Volume Ⅱ of the National Pharmaceutical Standards for Traditional Chinese Medicine Preparations by the former Ministry of Health) is implemented. |
| Wuwe Kushen Changrong Jiaonang | Kushen.*Sophorae Flavescentis Radix*  Diyu. *Sanguisorbae Radix*  Qingdai. *Baphicacanthus cusia (Nees) Bremek, Polygonum tinctorium Ait, Isatis indigotica Fort.*  *Baiji. Bletillae Rhizoma (dried rhizome of Bletilla striata (Thunb.) Reichb.f., Orchidaceae)*  Gancao. *Glycyrrhiza uralensis Fisch.* | YBZ00152015 (Standard of the National Medical Products Administration) |
| Yunnan Baiyao | Sanqi. *Panax notoginseng (Burk.) F.H. Chen* | The current official pharmaceutical standard for Yunnan Baiyao Capsules is stipulated in Volume I of the Pharmacopoeia of the People's Republic of China (2025 Edition). |

Note: The manufacture of the drug strictly complies with the quality standards stipulated in the Pharmacopoeia of the People's Republic of China (2020 Edition / 2025 Edition), and has obtained the "Guoyao Zhunzi" approval number issued by the National Medical Products Administration (NMPA).

**4 Supplementary Table S3 Adverse events to the included articles**

Table S3

| study | intervention | Adverse reaction | | |
| --- | --- | --- | --- | --- |
|  |  | T | C | |
| Wenqiu Bi, 2020 | T: WM_TCMD_acupuncture  C: WM | Hematochezia:2(35)  Intestinal stricture:0(35) | Hematochezia:5(35)  Intestinal stricture:3 (35) | |
| Shujun Chen,2017 | T: WM+TCMD  C: WM | Rash:1(50)  Fatigue:1(50)  Recurrence at 12-month follow-up:1(50) | Rash:1(50)  Elevated transaminase levels:1(50)  Decreased white blood cell count:1(50)  Recurrence at 12-month follow-up:8(50) | |
| Hu Ding et al., 2018 | T: WM+TCMD  C: WM | Recurrence at 6-month follow-up:2(45) | Recurrence at 6-month follow-up:8(45) | |
| Yang Fu, 2019 | T: WM+TCMD  C: WM | Dizziness:1(50)  Nausea:1(50) | Dizziness:2(50)  Nausea:3(50)  Constipation:3(50)  Rash:2(50) | |
| Xiaojun Lu, 2023 | T: WM+TCMD+ACE  C: WM/ TCMD+ACE | Nausea and vomiting: 2(34) | WM:  Nausea and vomiting:2(31)  abdominal pain：1（31）  Fatigue：1（31）  Decreased white blood cell count:1(31) | TCMD+ACE:  Nausea and vomiting:2 (34) |
| Liang Huang, 2024 | T: WM+TCMD  C: WM | Rash:3(50)  Nausea and vomiting: 1(50) | Rash:1(50)  Nausea and vomiting: 1(50)  Thrombocytopenia:1(50) | |

Table S3 (cont.)

| study | intervention | Adverse reaction | | |
| --- | --- | --- | --- | --- |
|  |  | T | C | |
| Caitang Liu, 2024 | T: WM+TCMD  C: WM | Gastrointestinal reactions:2(51)  Arthralgia:1(51) | Gastrointestinal reactions:3(51)  Arthralgia:1(51)  Headache:1（51） | |
| Longsheng Lu, 2010 | T: WM+TCMD  C: WM/TCMD | Mild adverse reactions：4（30）  Moderate adverse reactions：1(30) | WM：  Mild adverse reactions：4（30）  Moderate adverse reactions：2(30) | None |
| Yu Wang, 2019 | T: WM+TCMD  C: WM | Mild liver function abnormality:1(42) | Mild liver function abnormality:2(42) | |
| Zanren Wu, 2017 | T: WM+TCMD  C: WM | Recurrence at 6-month follow-up:2(30) | Nausea and vomiting: 1(50)  Recurrence at 6-month follow-up:5(30) | |
| Lihua Yu, 2022 | T: WM+TCMD  C: WM |  |  | |
| Xianqiong Zhang, 2022 | T: WM+TCMD  C: WM | Anal tenesmus:1(30) | Nausea:1(30)  Abdominal distension:2(30) | |
| Zhen Zhang, 2018 | T: WM+TCMD  C: WM | None | None | |

**Supplementary Figures: Consistency and Inconsistency of Outcome Indicators**

1. **Effective rate**

**
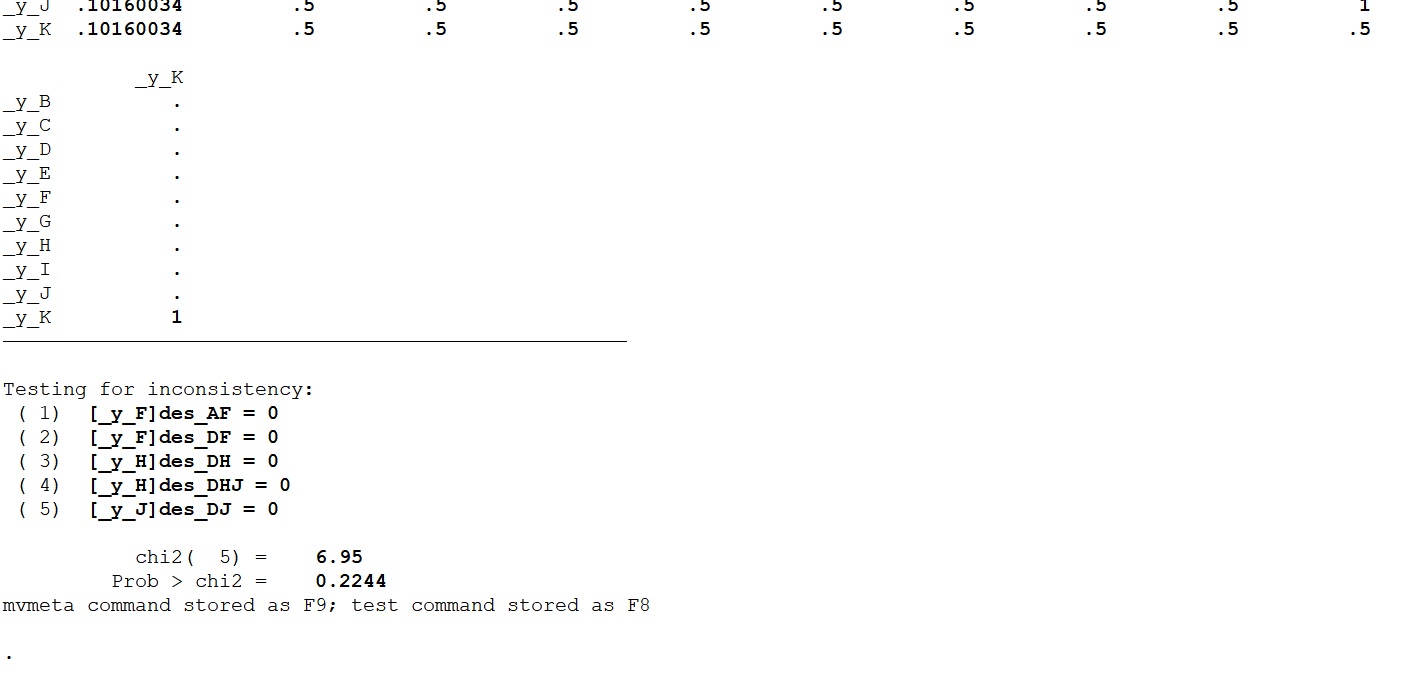
**

1. **Diarrhea**

**
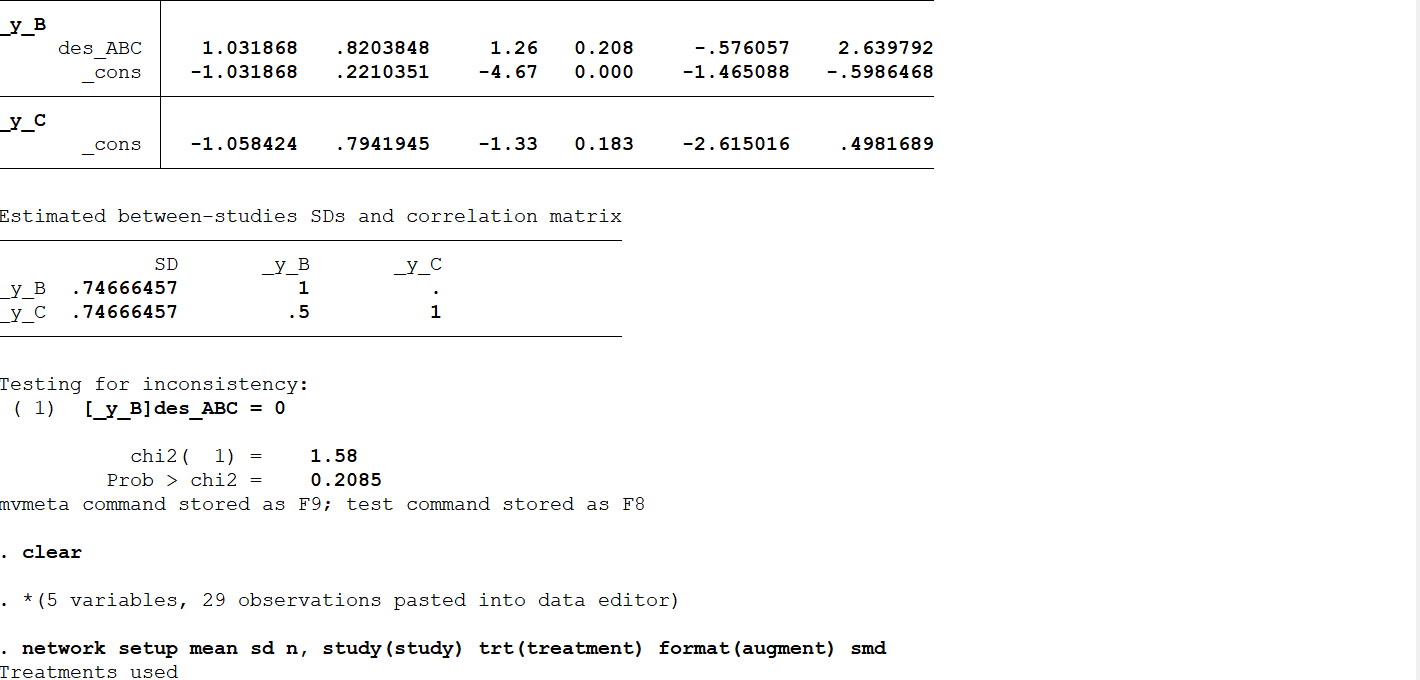
**

1. **abdominal pain**

**
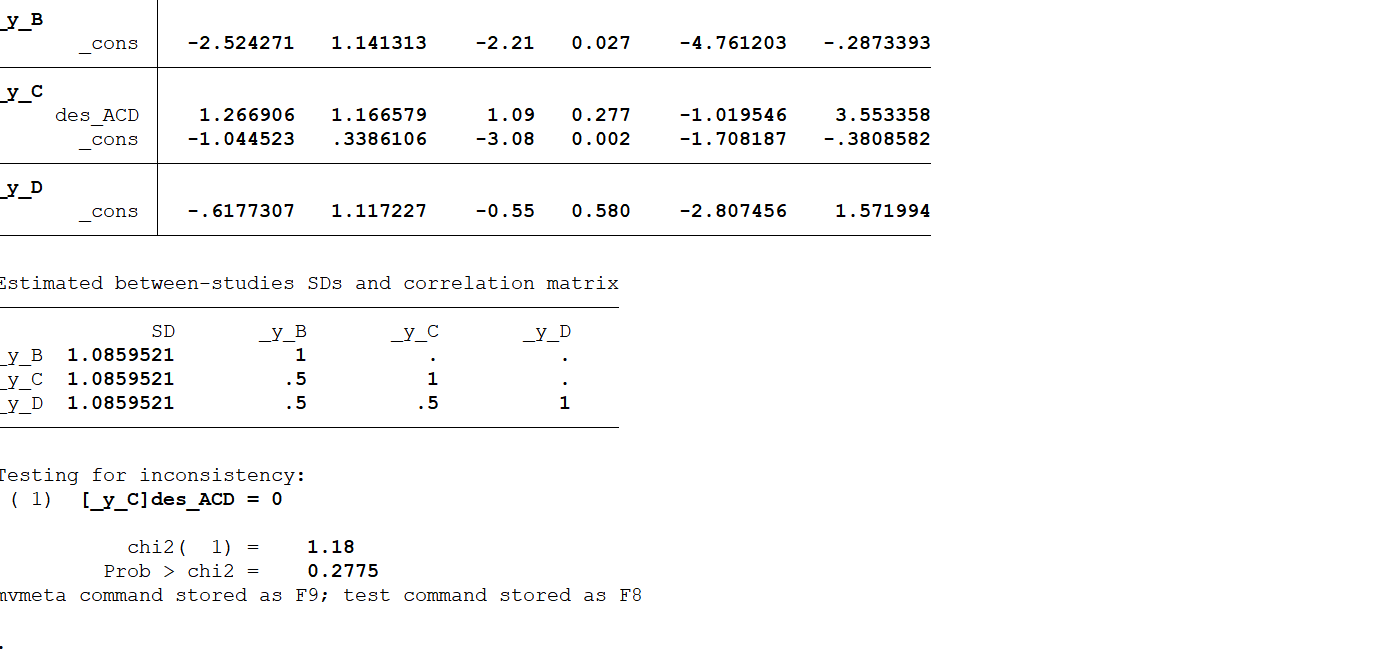
**

1. **Hematochezia**

**
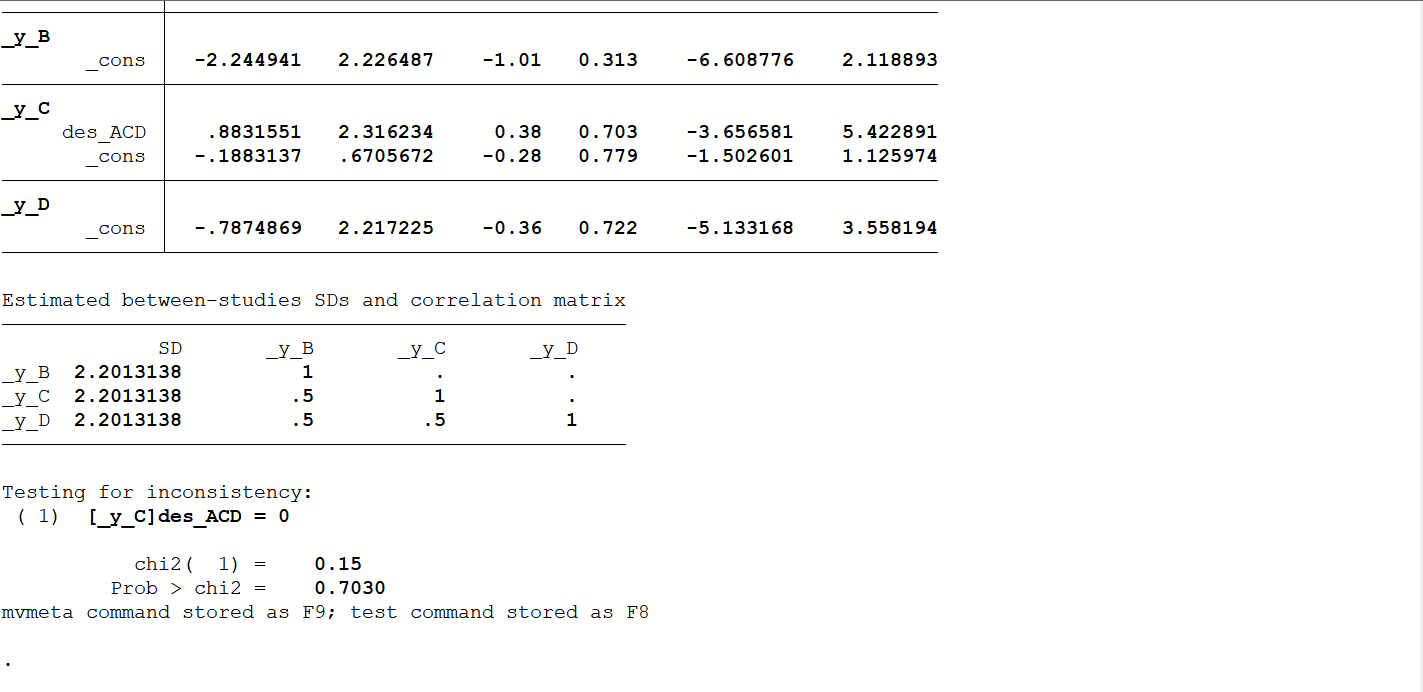
**

1. **Tenesmus**

**
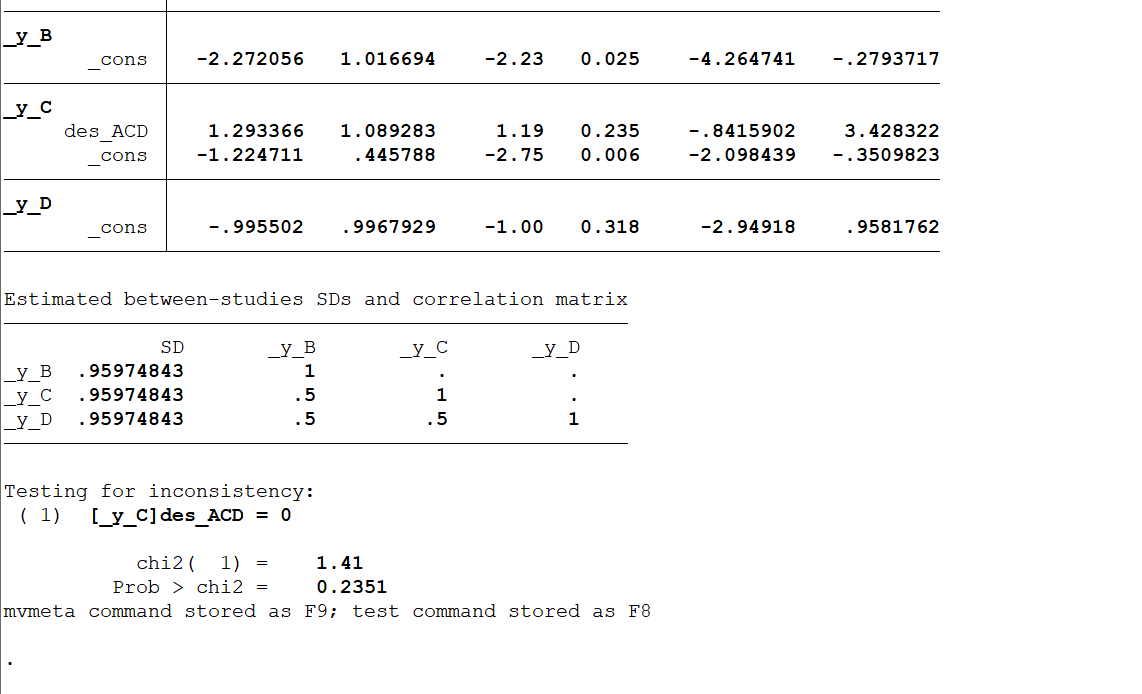
**

1. **C-reactive protein**

**
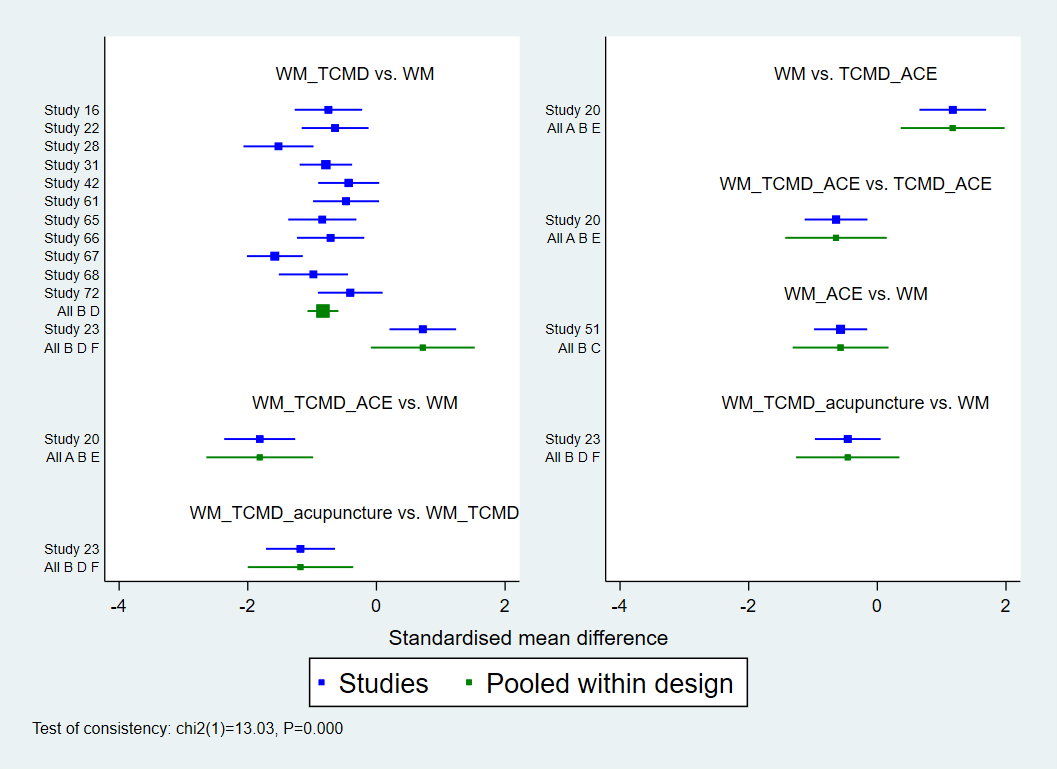
**

1. Erythrocyte Sedimentation Rate


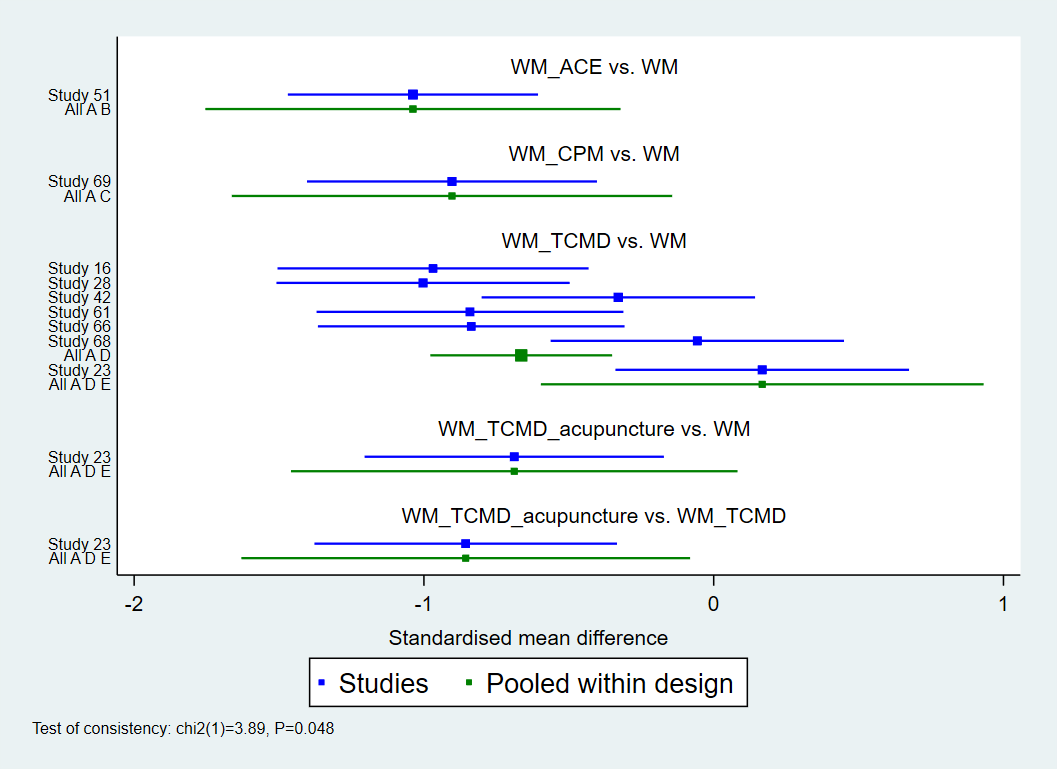


1. TNF-α


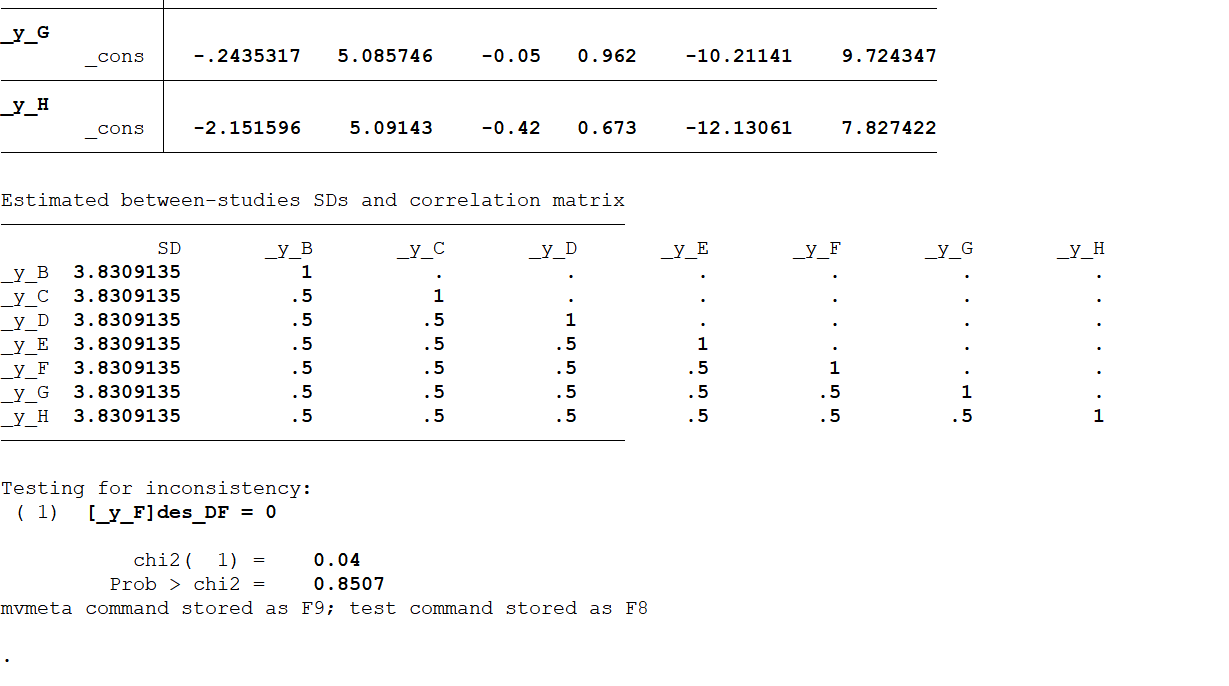


1. IL-6


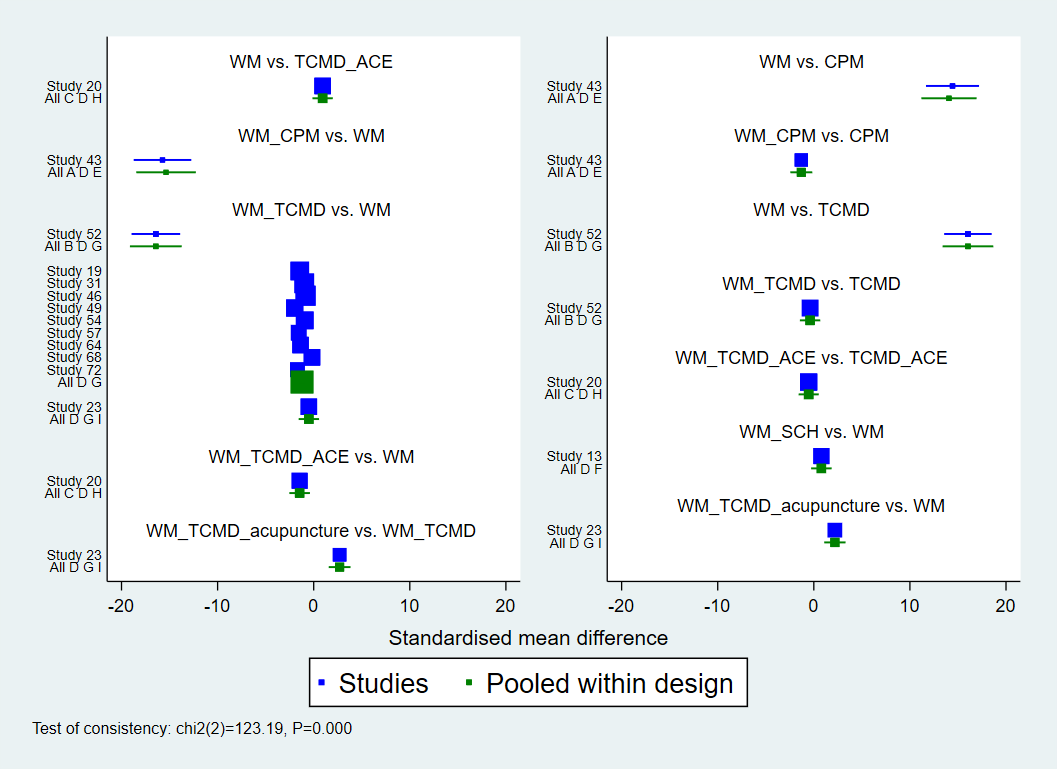


1. IL-8


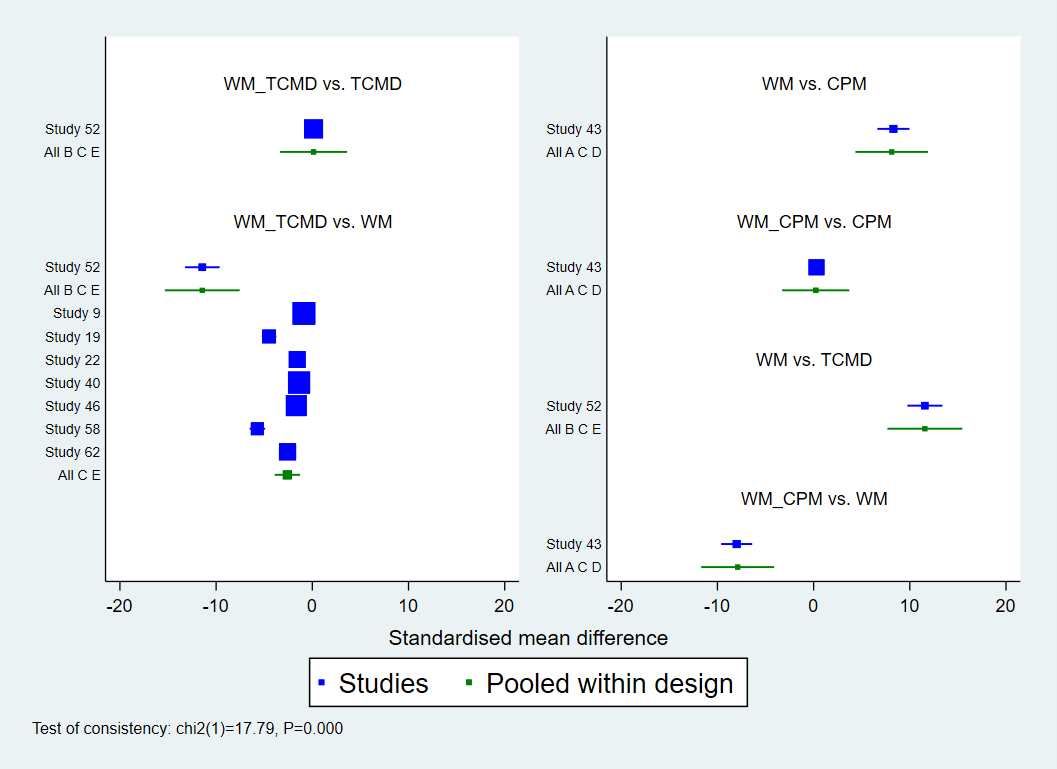


1. L-10


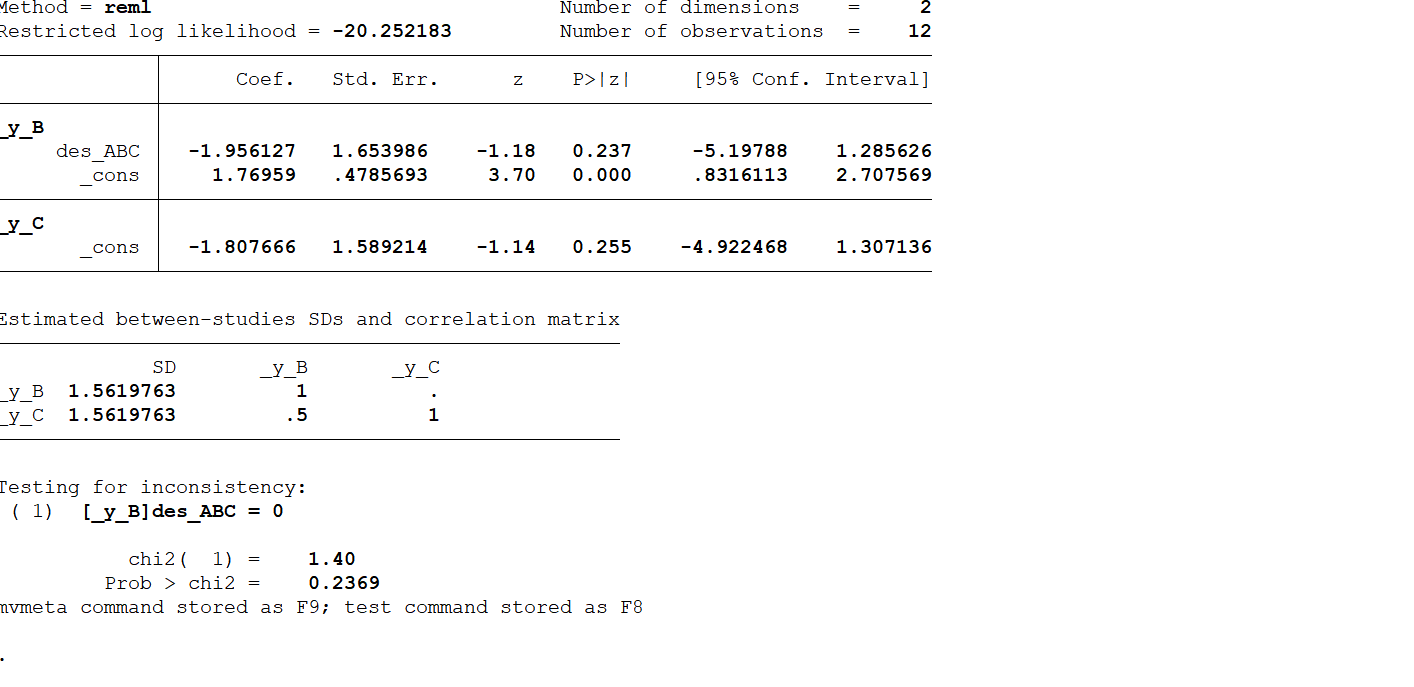

Supplement: Supplementary file 1 [file Supplementaryfile1.docx]
